# Molecular Mechanisms of Age‐Related Taste Dysfunction Deciphered by Spatiotemporal Transcriptomics

**DOI:** 10.1111/acel.70674

**Published:** 2026-08-20

**Authors:** Wandong Zhao, Xudong Cha, Yingqi Xie, Enhao Wang, Wenlu Xing, Zengyi Xu, Langchao Liang, Changhai Xiong, Morong Zhou, Penghui Fu, Zhe Wang, Xin Liu, Yiqun Yu, Huanhai Liu, Hanbo Li, Wenwen Ren

**Affiliations:** ^1^ College of Life Sciences University of Chinese Academy of Sciences Beijing China; ^2^ BGI Research Qingdao China; ^3^ BGI Research Shenzhen China; ^4^ Department of Otolaryngology The Second Affiliated Hospital of the Naval Military University (Shanghai Changzheng Hospital) Shanghai China; ^5^ BGI Research Hangzhou China; ^6^ School of Biology and Biological Engineering South China University of Technology Guangzhou China; ^7^ Department of Otolaryngology, Ear, Nose and Throat Institute Eye, Ear, Nose and Throat Hospital, Fudan University Shanghai China; ^8^ Olfactory Disorder Diagnosis and Treatment Center Eye, Ear, Nose and Throat Hospital, Fudan University Shanghai China; ^9^ State Key Laboratory of Genome and Multi‐Omics Technologies BGI Research Shenzhen China

**Keywords:** aging, macrophage, organoids, taste, taste progenitor cell

## Abstract

Age‐related taste dysfunction impairs nutrition and quality of life, contributing to metabolic disorders and frailty in the elderly, yet its cellular and molecular basis remains poorly understood. By integrating single‐cell RNA and spatial transcriptomics sequencing, we systematically mapped murine taste bud aging across five developmental stages from neonatal to aged. Our findings reveal that age‐associated taste impairment is driven by the progressive depletion of stem/progenitor cell differentiation capacity, predominantly characterized in the posterior tongue, and mediated by inflamm‐aging responses and micro‐environmental alterations. Specifically, we identified macrophage infiltration and polarization shifts in aged circumvallate and foliate papillae, underscoring the crucial role of macrophages in age‐related taste decline. A co‐culture model using taste organoids with conditioned media from polarized macrophage revealed that M1‐polarized cells suppress taste organoid proliferation and differentiation. Mechanistically, this inhibition may be mediated by macrophage‐derived Thbs1, as recombinant Thbs1 treatment directly suppressed taste progenitor cell expansion in vitro. Together, this study provides the first comprehensive spatiotemporal atlas of taste bud aging and identifies novel therapeutic targets for the treatment of age‐related taste disorders.

## Graphical abstract

Multi‐omics profiling reveals that aging impairs stem/progenitor cell differentiation and taste bud regeneration in the posterior tongue. Age‐related macrophage polarization remodeling, marked by the accumulation of pro‐inflammatory M1 macrophages, contributes to a Thbs1‐enriched inhibitory niche that limits taste cell growth and differentiation.

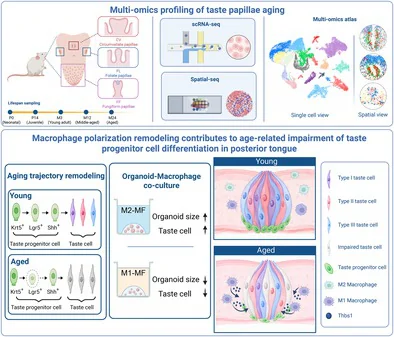

## 1 Introduction

Taste perception serves as an essential chemosensory system that profoundly impacts mammalian nutrition, health, and quality of life (Barlow 2022). Age‐related taste dysfunction represents a significant yet understudied geriatric condition, contributing not only to reduced dietary intake and life satisfaction but also to serious health complications including malnutrition and metabolic disorders (Feng et al. 2014). While the association between aging and taste impairment is well‐established, the precise cellular mechanisms driving this sensory deterioration remain poorly understood.

Our previous work identified age‐related molecular signature alterations in taste cells using single‐cell transcriptomics (Ren et al. 2024). Building on this, we now address the critical, unanswered questions concerning the spatiotemporal dynamics and mechanisms of taste bud aging. Although behavioral studies confirmed taste sensitivity decline in aged mice, previous technological constraints limited our ability to resolve the micro‐environmental changes underlying this functional decline. To overcome these limitations, we developed an innovative multi‐omics platform integrating high‐resolution single‐cell RNA sequencing (scRNA‐seq) with cutting‐edge spatial transcriptomics (Wei et al. 2022). Our comprehensive experimental design encompassed three functionally specialized taste papillae regions—circumvallate (CV), foliate (FL) of the posterior tongue, and fungiform papillae (FF) of the anterior tongue—across five critical developmental stages representing the murine lifespan: neonatal (P0), juvenile (P14), young adult (M3), middle‐aged (M12), and aged (M24) cohorts. This longitudinal design enabled unprecedented resolution of taste bud maturation and senescence while preserving critical spatial information about niche organization.

Our integrative analysis demonstrates that age‐related taste dysfunction emerges through two synergistic mechanisms, predominantly manifested in the posterior tongue: progressive depletion of taste stem/progenitor cell regenerative capacity and establishment of a pro‐inflammatory niche characterized by macrophage phenotypic switching, particularly through age‐dependent shift from M2‐like toward M1‐like state. These inflammatory macrophages may contribute to micro‐environmental alterations through the secretion of Thbs1, ultimately impairing stem/progenitor cell maintenance and differentiation. Furthermore, through computational prediction, we identified potential therapeutic targets among the differentially expressed genes in aged taste cells, providing novel candidates for treating age‐related taste disorders.

This study establishes the first spatiotemporal atlas of taste bud aging, providing comprehensive single‐cell profiling across five developmental stages and three tongue regions, refined classification of taste cell subtypes, and mechanistic insights into how inflammatory niche remodeling drives sensory dysfunction. Our findings not only advance fundamental understanding of chemosensory aging but also reveal actionable targets for therapeutic intervention in age‐related taste disorders.

## 2 Result

### 2.1 Cellular Atlas of Taste Papillae Through Single‐Cell and Spatial Transcriptomic Profiling

The murine lingual epithelium contains three principal types of gustatory papillae: CV and FL papillae in the posterior tongue and FF papillae distributed across the anterior region (Kumari and Mistretta 2023).

**FIGURE 1:**
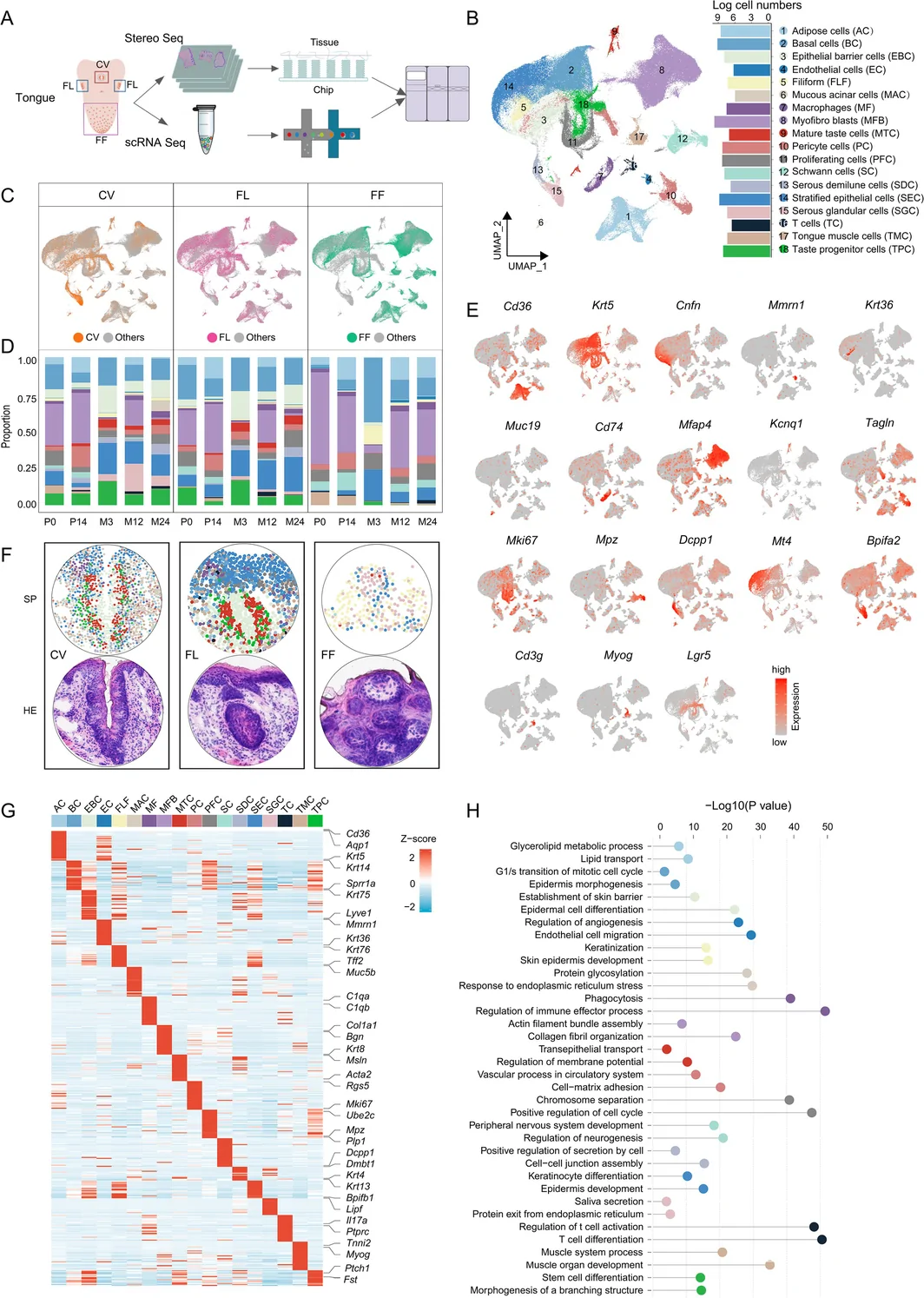
Single‐cell transcriptomic atlas of mouse taste papillae across aging. (A) Workflow for integrative analysis using scRNA‐seq and Stereo‐seq data. (B) Left: UMAP visualization of 18 cell clusters from integrated scRNA‐seq data. Right: Bar plot showing log‐normalized cell counts per cluster, colored by celltype annotation. (C) UMAP visualization showing cells from each taste papilla type highlighted in a unique color, with cells from the other papillae shown in gray. (D) Compositional bar chart showing cell‐type proportions across samples. (E) Gene expression plot displaying expression of key marker genes in each celltype, with gray for low and red for high expression. (F) Top: Spatial (SP) distribution of cells in representative taste bud regions. Bottom: Corresponding H&E‐stained histological sections from three taste papillae types. The color scheme used in all subpanels (A–F) is consistent with that shown in panel (B). (G) Heatmap of *z*‐score normalized expression for the top 30 marker genes per cell type. (H) Enriched GO biological processes for each cell population.

To systematically investigate transcriptomic dynamics in taste cell development across the lifespan, we conducted scRNA‐seq on CV, FL, and FF papillae at five developmental timepoints: postnatal day 0 (P0), postnatal day 14 (P14), 3 months (M3), 12 months (M12), and 24 months (M24), capturing the full spectrum of taste bud ontogeny and senescence. We also employed spatial transcriptomics (Stereo‐Seq) on tissue sections at P14, M3, M12, and M24. The integrated workflow was outlined in Figure 1A and Figure S1A.

Following quality control, 192,707 cells were retained for downstream analysis, among them, 83,542 cells from FF, 45,629 from CV, and 63,536 from FL (Table S1A). UMAP clustering revealed 18 cell populations (Figure 1B): Adipose cells (AC, *Aqp1* ^+^/*Cd36* ^+^) (Nicholls et al. 2011; Skowronski et al. 2016), Basal cells (BC, *Krt5* ^+^/*Krt14* ^+^) (Lu et al. 2022), Epithelial barrier cells (EBC, *Sprr1a* ^+^/*Cnfn* ^+^) (Turksen and Troy 2002; Wagner et al. 2019), Endothelial cells (EC, *Mmrn1* ^+^/*Lyve1* ^+^) (Becker and Wilting 2024), Filiform (FLF, *Krt36* ^+^/*Krt24* ^+^) (Brychtova et al. 2020), Mucous acinar cells (MAC, *Muc5b* ^+^/*Muc19* ^+^) (X. Wu et al. 2011), Macrophages (MF, *Cd74* ^+^/*C1qa* ^+^), Myofibroblasts (MFB, *Mtap4* ^+^/*Bgn* ^+^), Mature taste cells (MTC, *Trpm5* ^+^/*Kcnq1* ^+^), Pericyte cells (PC, *Tagln* ^+^/*Myl9* ^+^), Proliferating cells (PFC, *Mki67* ^+^/*Top2a* ^+^), Schwann cells (SC, *Plp1* ^+^/*Mpz* ^+^) (Shy et al. 2003), Serous demilune cells (SDC, C*lu* ^+^/*Ltf* ^+^), Stratified epithelial cells (SEC, *Krt13* ^+^/*Mt4* ^+^) (Miura, Kusakabe, et al. 2014; Xiang et al. 2025), Serous glandular cells (SGC, *Sval2* ^+^/*Bpifa2* ^+^), T cells (TC, *Cd3g* ^+^/*Cd69* ^+^), Tongue muscle cells (TMC, *Myog* ^+^/*Pax7* ^+^) and Taste progenitor cells (TPC, *Lgr5* ^+^/*Ptch1* ^+^) (Yee et al. 2013). The right panel of Figure 1B illustrated the logarithmic distribution of cell quantities across populations. Integration of single‐cell data with spatial data enabled high‐resolution spatial annotation of cell populations across heterogeneous tissue regions (Figure S1B).

**FIGURE 2:**
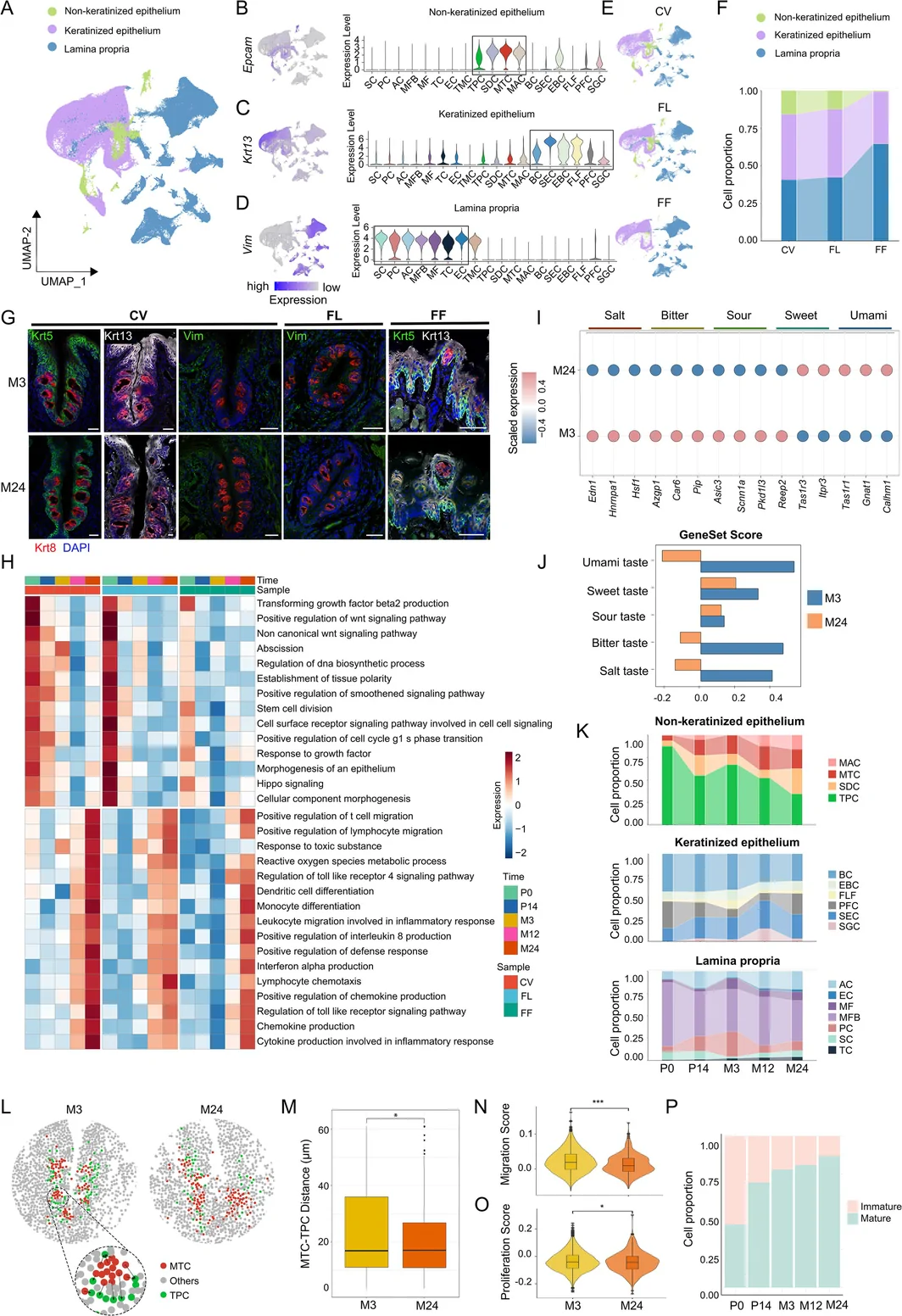
Comparative analysis reveals cell‐type and niche signatures underlying age‐related taste function decline. (A) UMAP showing the distribution of three tissue groups. (B–D) Left: Feature plots of compartment‐specific gene expression (*Epcam*, non‐keratinized epithelium; *Krt13*, keratinized epithelium; *Vim*, lamina propria). Right: Violin plots showing expression levels across cell types. (E) UMAP visualization of tissue group distributions in CV, FL, and FF. (F) Stacked bar plot showing the cell‐type composition of the three tissue groups. (G) Confocal microscopy images of CV, FL and FF papillae at M3 and M24 time points. Tissues were stained for basal keratinocytes (Krt5), pan‐gustatory cells (Krt8), squamous epithelial cells (Krt13) and lamina propria (Vim). No significant structural alteration was observed with aging. (H) Representative GO term score of CV, FL, and FF across different time points. (I) Normalized expression of taste‐associated genes at M3 and M24. (J) Bar plot showing taste related GO term scores at M3 and M24. (K) Temporal changes in cellular composition of three tissue groups. (L) Spatial distribution of MTC and TPC at M2 and M24. The cascaded magnification images showing quantification of MTC‐TPC distance based on Euclidean distance to nearest neighbor, arrows indicate the directions from each MTC to its nearest TPC. (M) Box plot illustrating significantly reduced MTC‐TPC distances at M24 compared to M3. (N, O) Violin plot indicating decreased migration and proliferation scores in TPC at M24. (P) Bar plot showing age‐dependent reduction in the proportion of immature MTC. *p < 0.05, ***p < 0.001 (two‐tailed Student's *t*‐test). Scale bar, 50 μm.

**FIGURE 3:**
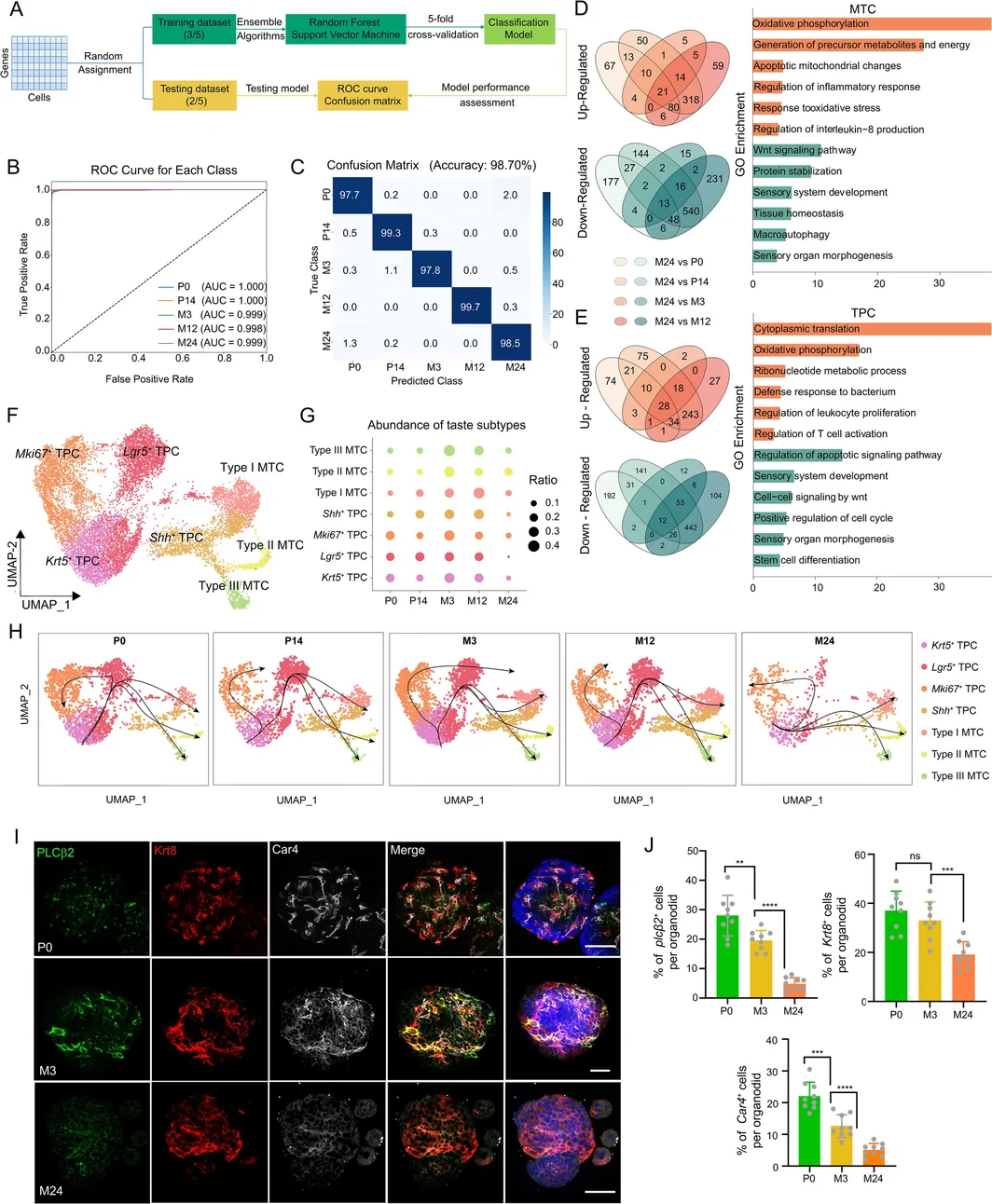
Gene expression alterations and impaired regeneration of taste cells with aging. (A) Diagram showing flowchart of taste cell classification analysis with machine learning. The dataset was randomly split into training and test sets at a 3:2 ratio. The model was trained with 5‐fold cross validation, and test dataset was used to evaluate the performance of model. (B) ROC curves indicating model accuracy across P0, P14, M3, M12 and M24 time points. Higher AUC values reflect improved ability to classify the time points. (C) Confusion matrix showing the distribution of prediction errors across time points. Color gradient (blue) represents classification proportion (darker = higher proportion). Overall accuracy = 98.70%. (D, E) Left: Venn diagrams showing overlap of DEGs from pairwise comparisons between M24 and other time points for MTC and TPC (orange, upregulated; green, downregulated). Right: Enriched GO terms among all up‐ (orange) and down‐regulated (green) DEGs. (F) UMAP plot showing the subclustering of MTC and TPC. (G) Dot plot depicting taste cell subtype abundance across the five time points, dot size corresponds to subtype proportion. (H) Pseudotime trajectories illustrating differentiation dynamics of taste cell subtypes from P0 to M24. Black curves represent inferred developmental paths. (I, J) Confocal images and quantification of PLCβ2 (Type II MTC marker), Krt8 (pan MTC marker) and Car4 (Type III MTC marker) signal intensities in taste organoids derived from P0, M3 and M24 aged mice. ns, not significant; **p < 0.01, ***p < 0.001, ****p < 0.0001 (unpaired *t*‐test). Scale bars, 50 μm.

**FIGURE 4:**
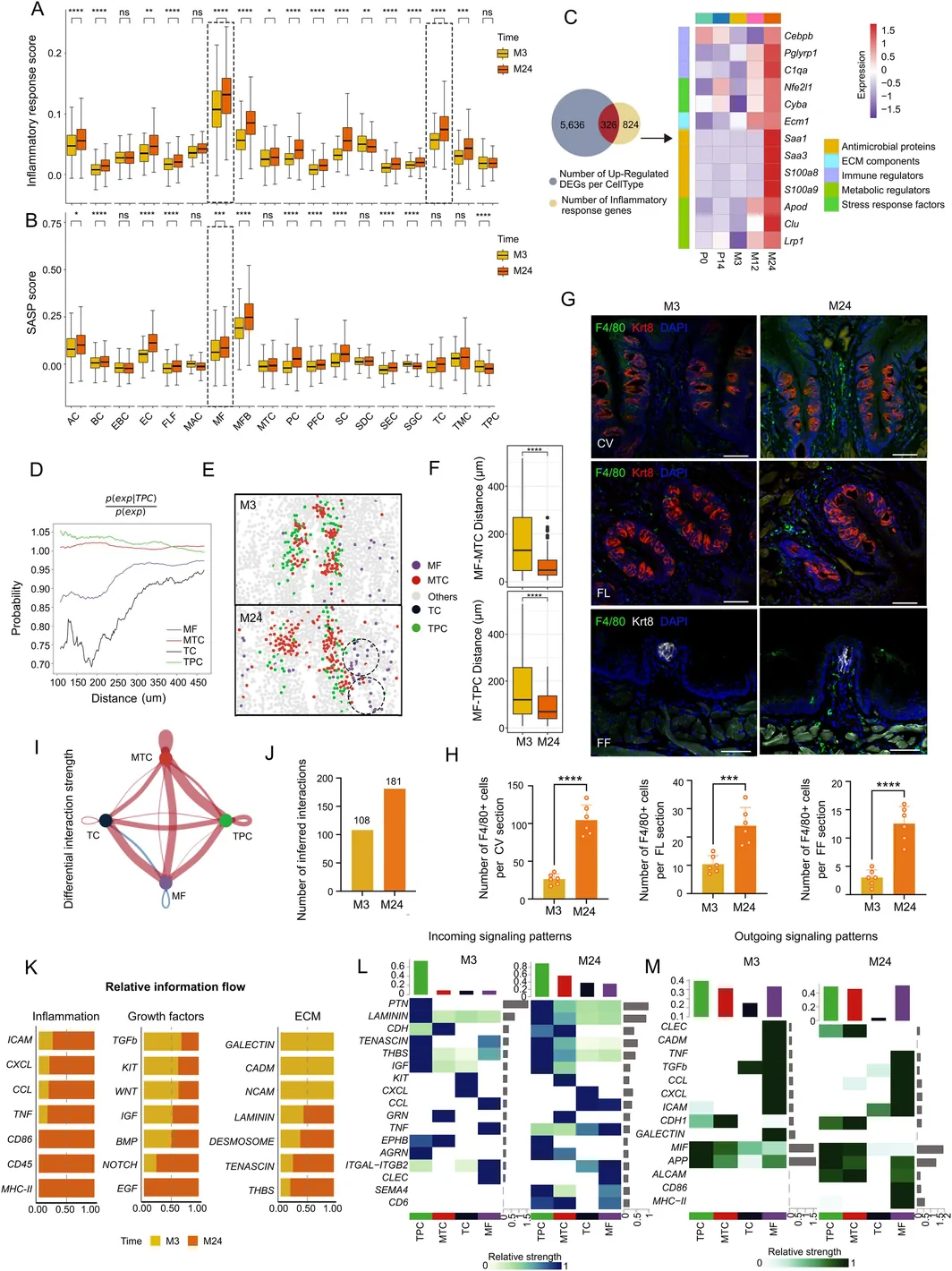
Inflamm‐aging enhances intercellular communication between MF and taste cells. (A, B) Gene set score for inflammatory response and SASP across cell populations. Most cell types exhibit higher scores at M24 (red) than M3 (orange) time points. Immune cells (dashed boxes) showing significant stage‐dependent differences. (C) Left: Venn diagram showing the number of overlapping genes between M24‐upregulated DEGs per cell type and the inflammatory response gene set from (A). Right: Heatmap of key intersection genes with elevated expression across all cell types at M24. (D) Co‐localization analysis showing the co‐occurrence probability of taste cells and immune cells within whole CV at M24. MF exhibits higher co‐localization probability with TPC than with TC at shorter distances. (E) Distribution of taste and immune cells in taste bud concentrated regions of CV papillae at M3 and M24. Black arrowheads indicate rare T cells. (F) Box plots showing significantly decreased distances from MF to MTC (top) and TPC (bottom) at M24 relative to M3. Distance metrics follow Figure 2L. (G, H) Confocal images and quantification of F4/80 (macrophage marker) and Krt8 (pan MTC marker) signal intensities in CV, FL and FF papillae at M3 and M24. *n* = 6 sections per group. Sections were collected from 3 mice per age group. (I) Network visualization of differential interaction strength between M3 and M24. Nodes represent cell types; red edges indicate M24 > M3, blue M24 < M3; thickness reflects differential interaction strength. (J) Bar plot of total interaction count, elevated at M24 compared to M3. (K) Stacked bars showing relative information flow for pathways grouped into inflammation, growth factors, and ECM categories. (L, M) Heatmaps of the incoming (L) and outgoing (M) signaling patterns for taste and immune cells at M3 (left) and M24 (right). *p < 0.05; **p < 0.01; ***p < 0.001; ****p < 0.0001; ns, not significant (two‐tailed Student's *t*‐test for B, unpaired *t*‐test for H). Scale bars, 50 μm.

**FIGURE 5:**
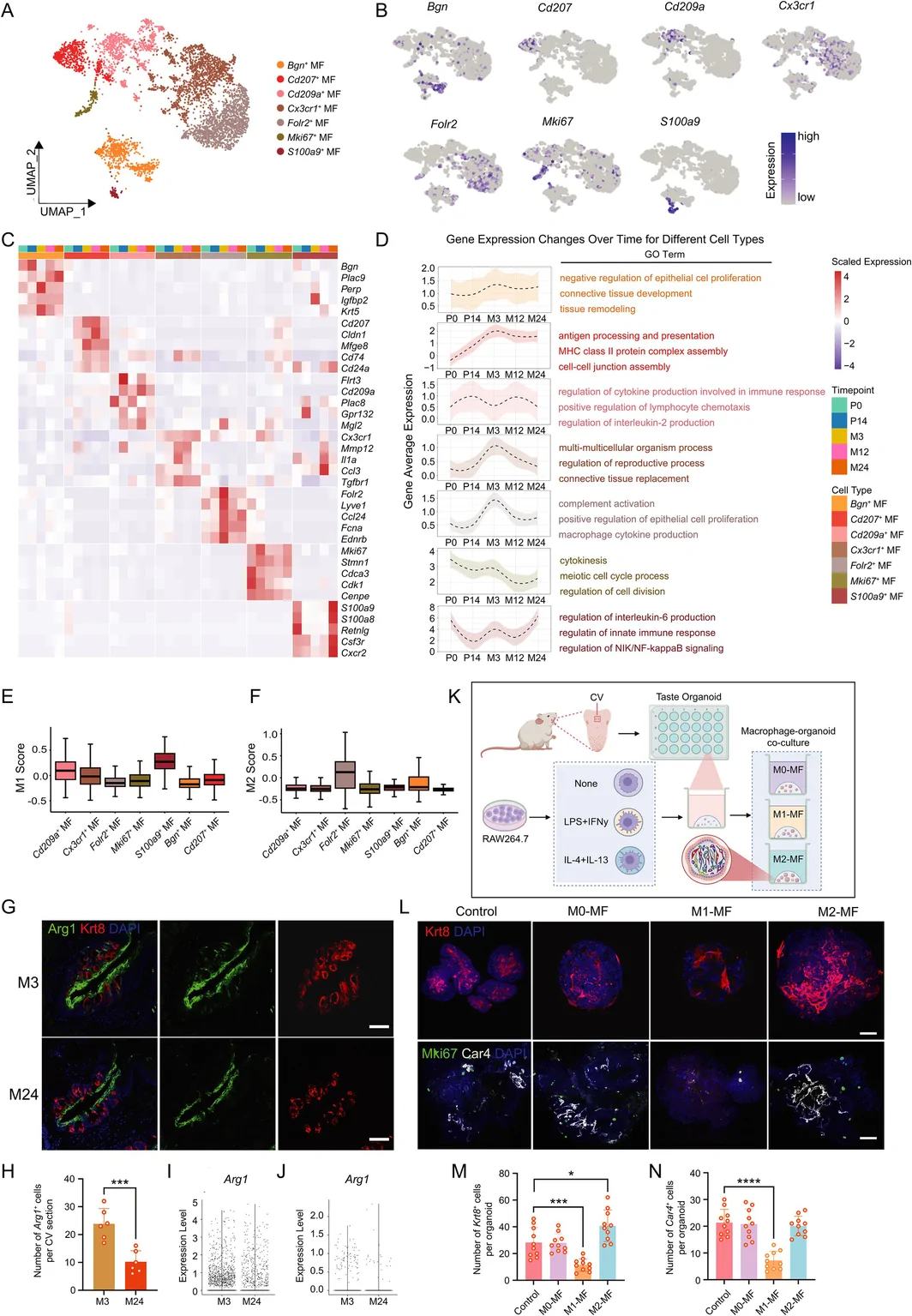
Aging disrupts macrophage polarization homeostasis and impairs taste cell regeneration. (A) UMAP visualization of MF subclustering. (B) Expression patterns of molecular markers for specific MF subtypes; gray indicates low expression, and purple gradients denote elevated expression. (C, D) Heatmap showing age‐associated expression changes of key DEGs across MF subtypes. Line plots illustrate expression dynamics of key DEGs within corresponding subtypes; dashed lines indicate mean expression, and shaded areas representing 95% confidence intervals. GO terms on the right indicate enriched biological processes for each specific subtype. (E, F) Box plots of M1‐like and M2‐like polarization scores across MF subtypes at M24. (G, H) Immunofluorescence images (Arg1, Krt8, DAPI) and quantification of Arg1^+^ cells in CV from M3 and M24 mice. (I, J) *Arg1* expression levels among all celltypes and taste cells at M3 and M24. (K) Flowchart of Macrophage‐ co‐culture system. RAW264.7 macrophages were polarized into M0‐, M1‐, or M2‐like states, and their conditioned media were applied to CV‐derived taste organoids. Created in https://BioRender.com. (L) Immunofluorescence staining of taste organoids (cultured for14 days) co‐cultured with control medium or M0/M1/M2‐like macrophage‐conditioned media, labeled with Krt8, Mki67, Car4 and DAPI. (M, N) Quantification of Krt8 and Car4 per organoid under each treatment condition. *p < 0.05, ***p < 0.001, ****p < 0.0001 (unpaired *t*‐test). Scale bars, 50 μm.

**FIGURE 6:**
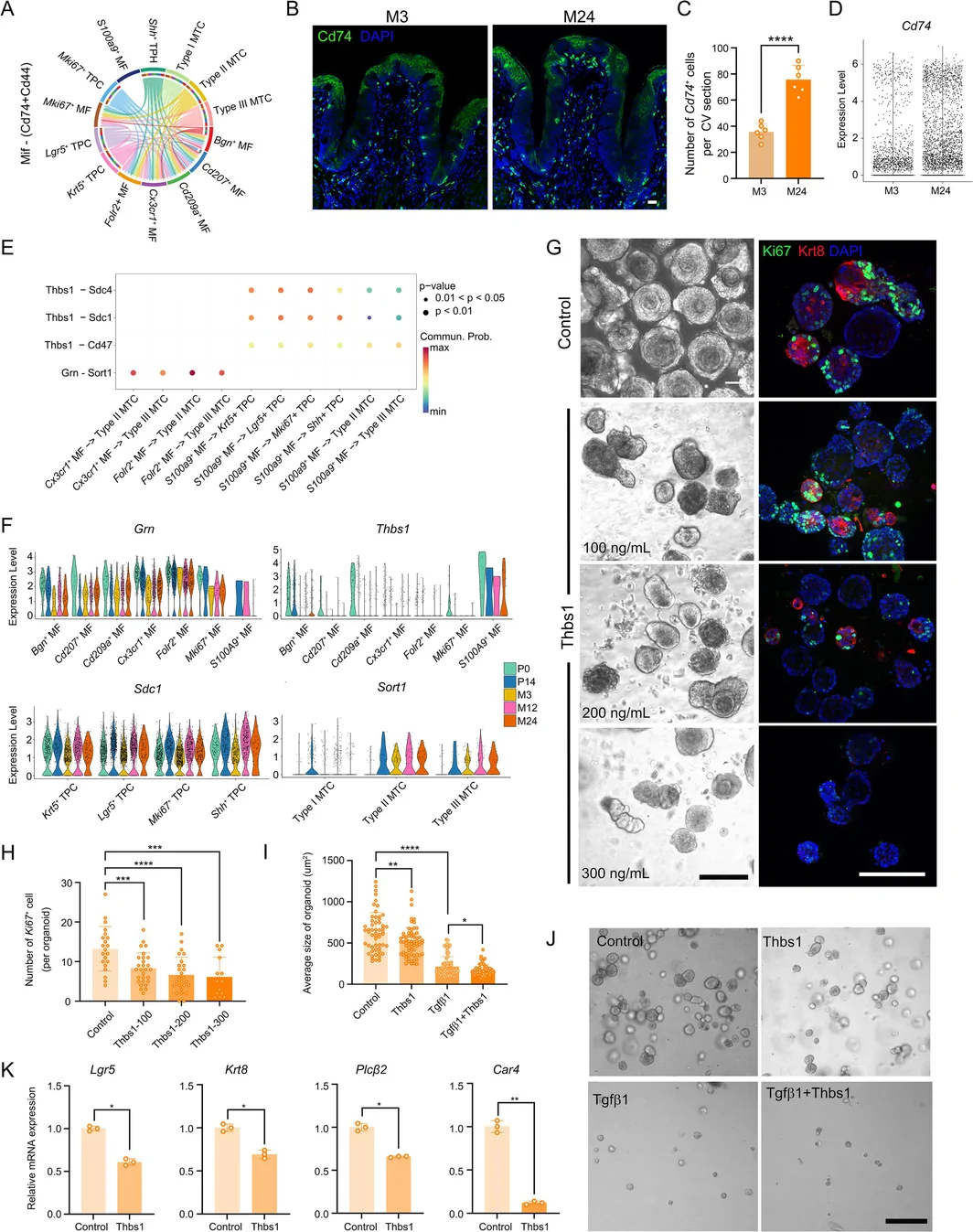
Thbs1‐mediated macrophage–taste cell crosstalk suppresses taste organoid proliferation and differentiation. (A) Chord diagram depicting Mif‐(Cd74 + Cd44) signaling interactions between taste cell and MF subtypes at M24; ribbon color and thickness reflects interaction specificity and strength, respectively. (B, C) Immunofluorescence images and quantification of Cd74 in CV from M3 and M24 mice. (D) Expression level of *Cd74* in MF at M3 and M24. (E) Dot plot of key ligand‐receptor pairs underlying MF to taste cell subtypes interactions at M24. (F) Violin plot showing age‐related expression trends of key ligands (in MF subtypes) and corresponding receptors (in taste cell subtypes) across developmental stages. (G) Bright‐field and immunofluorescence images of taste organoids (cultured for 9 days) treated with recombinant Thbs1 at different concentrations. Organoids were stained for Ki67, Krt8, and DAPI. (H) Quantification of Ki67^+^ cells per organoid after Thbs1 treatment. (I, J) Quantification of organoid size and representative bright‐field images of taste organoids (cultured for 6 days) treated with Thbs1, Tgfβ1, or their combination. (K) RT‐qPCR analysis of mRNA expression levels of *Lgr5*, *Krt8*, *Plcβ2*, and *Car4* in taste organoids (cultured for 9 days) after Thbs1 treatment (100 ng/mL). *n* = 3 independent experiments per group. *p < 0.05, **p < 0.01, ***p < 0.001, ****p < 0.0001 (unpaired *t*‐test). Scale bars, 100 μm.

**FIGURE 7:**
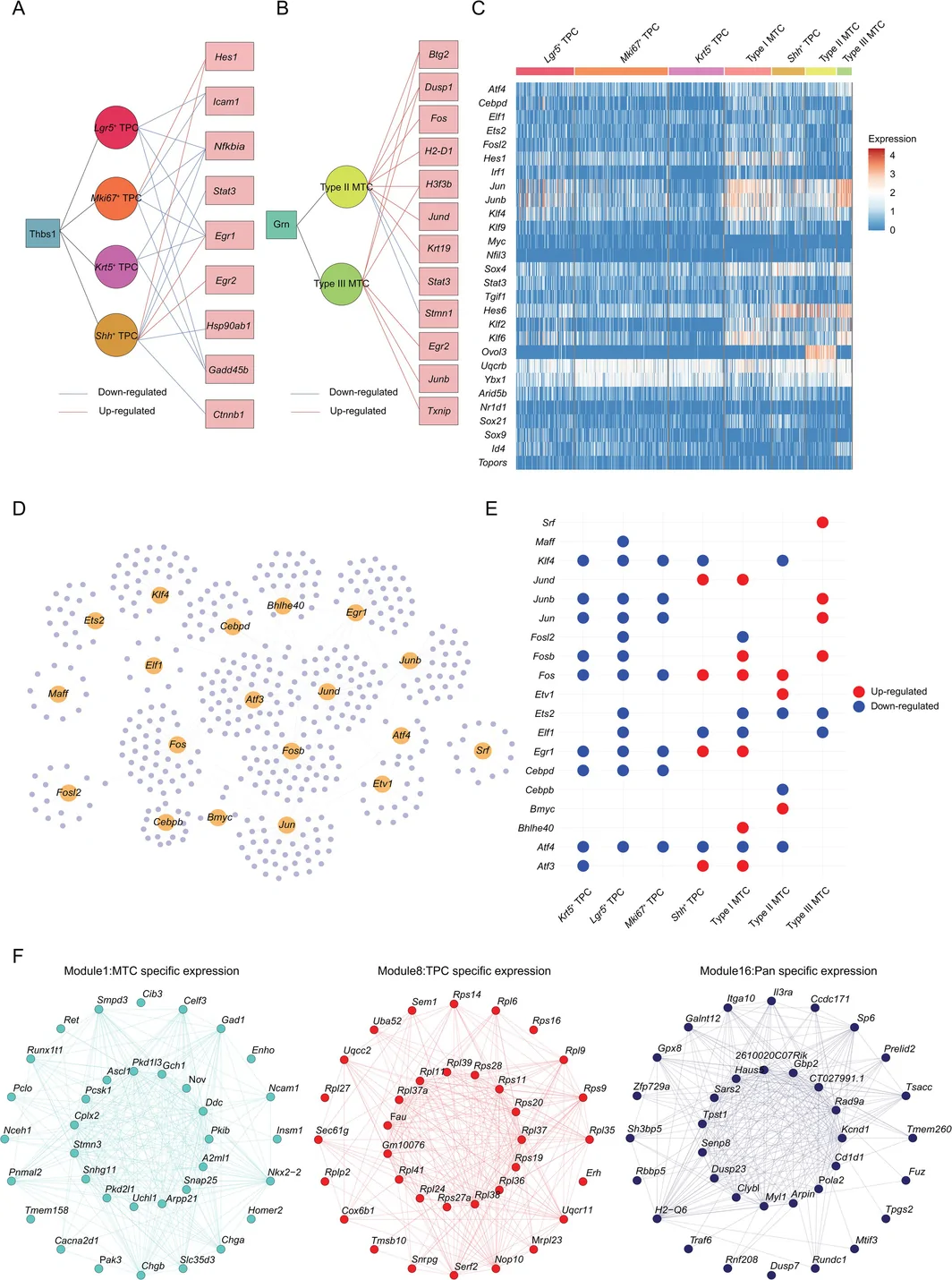
Age related master regulators in the transcriptional network of taste cells. (A, B) Regulatory networks of key ligand‐mediated interactions in taste cell subtypes at M24. Orange edges represent upregulated interactions and blue edges indicate downregulation. (C) Heatmap showing expression of TFs with significant shifts within taste cell subtypes in M24, identified by intersecting DEGs (|Log_2_FC| > 0.5, *p* < 0.05) with predicted TFs. (D) Visualization of the regulatory network among target genes controlled by TFs identified in (C). (E) Dot plot illustrating subtype‐specific up‐ and down‐regulation of selected TFs across taste cell subtypes in M24. Red indicates upregulation, blue represents downregulation. (F) Gene co‐expression networks highlighting modules 1, 8, and 16 in taste cells. Nodes represent genes; edges indicate co‐expression relationships. The top 15 hub genes (by connectivity) are shown in the center, surrounded by 20 peripheral genes.

Distinct cellular organization across papillae types was revealed by UMAP visualization and cell type proportion quantification (Figure 1C,D). Feature plots validated the expression of established marker genes for each identified cell population (Figure 1E). Spatial transcriptomic combined with HE staining enabled precisely mapped cellular localization, revealing distinct tissue architectures among CV, FL, and FF (Figure 1F). Expression of *Lgr5, Kcnq1*, *Krt5*, and *Mt4* in TPC, MTC, BC, and SEC, as well as *Krt24* (a marker enriched in filiform‐papilla‐region) in FLF was confirmed by spatial mapping and feature plots within CV, FL, and FF (Figure S1C–E). These patterns aligned with anterior–posterior regionalization, as von Ebner's glands were abundant in CV, with duct serving as a source of *Sox10* ^+^ progenitors (Yu et al. 2020; Yu et al. 2024), while FF papillae lacked *Lgr5* expression in adults (Ren et al. 2014; Takeda et al. 2013) and were surrounded by dense filiform projections, demonstrating a subtle yet significant gene expression difference. Given the sparse representation of TPC and MTC in the FF dataset (Table S1B,C), our subsequent analyses of cell‐type dynamics and molecular mechanisms during aging (Figures 2, 3, 4, 5, 6, 7) primarily focus on the taste‐rich posterior papillae (CV and FL).

Heatmap and GO enrichment analysis revealed cell‐type‐specific biological processes (Figure 1G,H). MTC was enriched in transepithelial transport and regulation of membrane potential; SGC was associated with saliva secretion and protein processing in the endoplasmic reticulum; TPC in stem cell differentiation and branching morphogenesis, consistent with their role in maintaining taste bud homeostasis. Thus, by integrating spatial and temporal dimensions, we provided a higher‐resolution categorization system for taste cell populations.

### 2.2 Age‐Related Decline in Taste Function Emerges From Progenitor Cell Depletion and Niche Dysregulation

Based on developmental origin, histological features, and functional specialization, we classified 18 identified cell populations into three principal compartments (Figure 2A–D).

The non‐keratinized epithelium, characterized by *Epcam* expression, encompasses taste‐associated lineages (MTC and TPC) and glandular secretory cells (SDC and MAC) that constitute the epithelial‐derived components of salivary glands (Figure 2B). The keratinized epithelium comprising six stratified squamous subtypes (BC, SEC, EBC, FLF, PFC, and SGC) marked by *Krt13*, forms a continuous keratinized barrier through terminal differentiation (Figure 2C). Unlike sensory epithelium, this compartment lacks taste buds and primarily serves protective functions via physical shielding and microbial defense (Iwasaki et al. 2006; Miura, Kusakabe, et al. 2014). The lamina propria, a subepithelial connective tissue matrix, includes seven stromal subtypes (SC, PC, AC, MFB, MF, TC, and EC) based on vimentin (*Vim*) expression (Figure 2D), containing nerve fibers and immune cells that support nutrient exchange, neural signaling, and microenvironment homeostasis (Mistretta and Kumari 2017; Ren et al. 2020). TMC was excluded from lamina propria despite being *Vim* ^+^. The distribution and proportions of these compartments were altered in the posterior tongue (CV and FL) versus anterior tongue (FF), where non‐keratinized epithelium was limited due to epithelial cell proportion in FF (Figure 2E,F). Immunofluorescence confirmed spatial distributions of the three cell groups (Figure 2G). Key markers included *Krt8* (pan‐mature taste cells) (Okubo et al. 2009), *Krt5* (basal keratinocytes), *Krt13* (squamous epithelial cells), and *Vim* (lamina propria). No significant age‐dependent changes in cellular localization were observed between young (M3) and aged (M24) mice, supported by the stable expression of key marker genes (*Krt8*, *Cdh1*, *Epcam*, *Krt5*, *Krt14*, *Krt13*, and *Vim*) across all time points (Figure S2A–C). Thus, the core cellular architecture of taste papillae remains stable throughout aging.

However, Gene Set Variation Analysis (GSVA) uncovered distinct biological process activities across the lifespan (Figure 2H). Pathways related to development, homeostasis, and differentiation (such as Wnt, Notch, Hippo, and Stem cell division) were highly expressed neonatally (P0), decreased postnatally, and reached the lowest level at M24, indicating reduced differentiation capacity (Ishan et al. 2023; Liu et al. 2007; Seta et al. 2003). In aged mice, immune and inflammatory responses were upregulated across all three taste papillae groups, aligning with their roles in environmental defense and immune cell recruitment. Notably, while both non‐keratinized and keratinized epithelium displayed increased immune activity by M24, the lamina propria exhibited elevated inflammation as early as M3, suggesting earlier immune recruitment in this region (Figure S2E,F).

These alterations in biological process affected taste function, as evidenced by behavior analysis showing that aged mice exhibited reduced sensitivity to several tastants (Ren et al. 2024). Accordingly, most genes associated with the perception of five basic tastes showed lower expression in M24 versus M3 mice (Figure 2I). Sensory perception scores further supported functional decline with age (Figure 2J and Table S2).

Quantification of cell type proportions across five time points revealed the most pronounced decline in TPC from M3 to M24 (Figure 2K), correlating with functional deterioration and consistent with downregulation of taste stem/progenitor markers (*Lgr5*, *Fst*) in aged mice (Ren et al. 2024).

To investigate the mechanisms underlying taste niche alterations during aging, we evaluated the spatial juxtaposition of MTC and TPC to examine the changes of their proximity. Representative images illustrated the distribution of MTC (red) and TPC (green) in M3 and M24 mice (Figure 2L). Quantification of the shortest Euclidean distances from each MTC to the nearest TPC, as shown in the enlarged view of the circled area (Wang, Wang, Liao, et al. 2025), revealed a significant age‐related decrease in MTC‐to‐TPC distance in CV tissues at M24 compared to M3 (*p* = 0.025) (Figure 2L,M). Moreover, stem cell proliferation capacity and TPC migratory potential were markedly reduced at M24 (Figure 2N,O; gene sets used for scoring are listed in Table S3). Since *Shh* ^+^ cells exhibit dual characteristics of taste precursor cells and immature taste cells (Miura, Scott, et al. 2014), we stratified MTC into immature (*Shh* ^+^) and mature (*Shh* ^−^) states. Notably, the proportion of *Shh* ^+^ immature taste cells within the MTC population progressively decreased with age (Figure 2P), suggesting a pronounced impairment in taste cell regenerative capacity in aged mice. Collectively, these findings pointed to potential age‐related disruptions in intercellular communication and/or niche maintenance mechanisms.

### 2.3 Aged Taste Precursor Cells Exhibit Reduced Differentiation Ability

To investigate gene expression changes in taste bud degeneration, we employed machine learning to classify aged taste cells by transcriptional profiles (Wang, Ge, Liao, et al. 2025). Using scRNA‐seq data across all stages, we implemented an ensemble learning algorithm to construct a predictive classification model. The dataset was randomly split into training (60%) and testing (40%) subsets, with model training performed exclusively on the training set. Model performance was rigorously evaluated on the independent test set using both Receiver Operating Characteristic (ROC) curve analysis and confusion matrices (Figure 3A).

The ensemble model demonstrated exceptional performance, achieving an average Area Under the Curve (AUC) of 0.99 across multiple iterations. Prediction accuracies ranged from 97.8% to 99.7%, underscoring the model's robust discriminatory power (Figure 3B,C). These results confirmed the existence of temporally regulated gene expression signatures that not only accurately identify M24‐stage taste cells but also effectively distinguish them from other developmental populations with near‐perfect precision. To assess age‐related degeneration, we compared DEGs in TPC and MTC across stages. The overlap of DEGs between M24 and other time points (Table S4) was visualized using Venn diagrams (Figure 3D,E). GO enrichment analysis revealed profound age‐associated dysregulation. In aged (M24) mice, both populations exhibited upregulation of oxidative phosphorylation and metabolic pathways (Table S4). MTC additionally upregulated inflammatory response and energy precursor production, while TPC displayed elevated cytoplasmic translation and antibacterial defense. Conversely, taste‐related pathways, particularly Wnt signaling, sensory system development, protein stabilization, and stem cell proliferation were downregulated, indicating a mechanistic imbalance underlying age‐related taste dysfunction. The sustained metabolic activation, coupled with the suppression of developmental and regenerative pathways, implied a compensatory response that ultimately fails to counteract declining sensory cell renewal and diminished signal transduction efficiency.

Pseudotime analysis of DEGs across five timepoints using Monocle2, coupled with functional enrichment, revealed dynamic transcriptional reprogramming of MTC and TPC during aging (Figure S3A,B). Unsupervised clustering analysis categorized the DEGs into six temporally distinct expression clusters. In MTC, clusters 2, 4, and 5 comprised genes consistently upregulated at M24, associated with apoptotic signaling regulation, cellular component disassembly, and inflammatory response modulation. Clusters 1, 3, and 6 comprised downregulated genes involved in developmental processes, including ATP metabolic process, oxidative phosphorylation, and intracellular transport. In TPC, clusters 1 and 5 contained age‐upregulated genes related to energy metabolism, apoptotic signaling, and protein folding, while remaining clusters exhibited downregulation of genes linked to developmental processes, protein stability, epithelial cell proliferation, and mitotic cell cycle phase transitions. Collectively, these findings highlighted a coordinated transcriptional shift from developmental programs to stress‐response pathways during aging, underscoring the distinct yet converging molecular trajectories of MTC and TPC over time.

Marker‐based clustering classified MTC and TPC into seven distinct subpopulations: *Krt5* ^+^, *Lgr5* ^+^, *Mki67* ^+^, and *Shh* ^+^ TPCs, along with Type I (*Nupr1* ^+^), Type II (*Gnat3* ^+^), and Type III (*Pkd1l3* ^+^) MTC (Figure 3F). Feature plots and dot plot confirmed marker specificity (Figure S3C,D). Quantitative analysis revealed a significant reduction in the abundance of all subpopulations at the M24 stage (Figure 3G), consistent with the age‐related decline in taste function. This broad depletion suggested a generalized impairment in both progenitor cell maintenance and mature cell homeostasis, contributing to taste dysfunction during aging.

Furthermore, Slingshot trajectory analysis uncovered fundamental alterations in the differentiation program with aging. Younger mice (P0‐M12) exhibited a canonical three‐step differentiation hierarchy (Krt5^+^ TPC→Lgr5^+^ TPC→Shh^+^ TPC→MTC), while aged mice (M24) displayed a simplified, direct differentiation trajectory bypassing the critical *Lgr5* ^+^ intermediate stage (Figure 3H). This remodeling correlated with our previous observation of age‐dependent *Lgr5* ^+^ TPC depletion (Ren et al. 2024), suggesting that loss of this key progenitor pool forces a compensatory but potentially less efficient differentiation shortcut.

To further validate the age‐related decline in the regenerative capacity of taste stem/progenitor cells, we established taste organoid cultures from P0, M3, and M24 aged groups. Immunostaining analyses demonstrated a substantial decrease in the generation of mature taste cells in aged organoids, compared to the other two groups, with marked reductions in both Type II (PLCβ2‐expressing) and Type III (Car4‐expressing) cell populations, as well as in cells expressing the pan‐taste cell marker Krt8 (Figure 3I,J, *p* < 0.001), suggesting that aging not only diminishes the pool of taste stem/progenitor cells but also impairs their differentiation potential into functional mature taste cells. The observed age‐related decline in both stem cell maintenance and lineage commitment may underlie the well‐documented deterioration of taste function in the elderly population.

### 2.4 Aged Taste Papilla Undergoes Inflamm‐Aging Along With Increased Macrophage Infiltration

Previous studies have identified inflammatory factor receptors in taste buds (Wang et al. 2009), suggesting a potential link to taste decline. To investigate the cellular basis of inflammatory responses during aging, we performed gene set enrichment analysis using an inflammatory response gene panel from MiSgDB (Table S5A). Immune cells, particularly macrophages (MF) and T cells, exhibited the highest inflammatory activity (Figure 4A). Given the role of senescence‐associated secretory phenotype (SASP) in aged tissues, we additionally scored cells using SASP‐related gene sets (Table S5B), further confirming MF as the most prominently altered cell type during aging (Figure 4B). Intersection analysis between M24‐upregulated DEGs and the inflammation‐related genes from Figure 4A identified 326 co‐expressed genes spanning immune regulators (*Cebpb*, *Pglyrp1*, *C1qa*), stress response factors (*Nfe2l1*, *Cyba*), ECM components (*Ecm1*), antimicrobial proteins (*Saa1/3*, *S100a8/9*), and metabolic regulators (*Apod*, *Clu*, *Lrp1*) (Figure 4C). All these genes showed elevated expression in aged tissues, suggesting a broad cellular response to inflammatory stimuli. Collectively, these findings positioned MF as a key mediator of inflamm‐aging, orchestrating pro‐inflammatory networks through SASP activation and coordinated expression of inflammatory mediators.

Co‐location analysis further revealed that MF exhibited significantly closer spatial proximity to both MTC and TPC compared to T cells, suggesting stronger functional interactions between MF and taste cell populations (Figure 4D). Quantitative spatial analysis, using the same calculation method as in Figure 2L, revealed an age‐dependent clustering pattern of MF around taste cells, with progressive accumulation of MF adjacent to MTC/TPC clusters in aged mice (Figure 4E,F).

This remodeling of the immune micro‐environment was further corroborated by immunofluorescence staining, showing markedly increased F4/80^+^ macrophage infiltration in the lamina propria of CV, FL, and FF in aged mice, compared to M3 stage (Figure 4G,H).

Comparative CellChat analysis revealed a significant age‐associated enhancement in total immune cell‐taste cell interactions, along with a higher number of inferred interactions in M24 stage (Figure 4I,J). Aged tissue exhibited pronounced enrichment of inflammation‐related pathways, growth factor signaling, and ECM‐mediated crosstalk (Figure 4K). Specifically, pro‐inflammatory input pathways (ICAM, CXCL, CCL, TNF, CD86, CD45, MHC‐II), growth factor‐related pathways (NOTCH, EGF), and ECM pathways (LAMININ, DESMOSOME, TENASCIN, THBS) were upregulated in M24‐aged mice, while regenerative signals (TGFb, KIT, WNT, GALECTIN, CADM, NCAM) were significantly diminished.

To further clarify intercellular communication dynamics, we analyzed input/output signaling patterns among four major cell types. Each population exhibited distinct signal reception profiles that correlated with specialized biological functions. TPC exhibited high reception intensities for LAMININ, TENASCIN, THBS, and IGF at both M3 and M24 stages (Figure 4L). The overall signaling intensity was markedly elevated at the M24 stage (Figure 4K), implying these pathways may regulate TPC function and age‐related processes. Incoming signals identified MF as the primary recipients of CCL, ITGAL‐ITGB2, GALECTIN, and TNF pathways. Outgoing signaling patterns varied substantially across cell types. MF stood out as prominent signal senders, robustly expressing CLEC, CADM, and TNF, underscoring their potential pivotal role in immune modulation and inflammatory responses through these secretory pathways (Figure 4M).

Collectively, these findings provided a comprehensive map of cellular communication networks and further indicated macrophages as key mediators of inflamm‐aging in taste buds, orchestrating molecular reprogramming of the taste epithelium during aging.

### 2.5 The Polarization Homeostasis of Macrophages Is Altered in Aged Taste Bud Tissues

To identify macrophage subtypes driving age‐related changes in taste tissue, we conducted high‐resolution subclustering of macrophages near taste papillae and uncovered seven transcriptionally distinct subtypes: *Bgn* ^+^ MF, *Cd207* ^+^ MF, *Cd209a* ^+^ MF, *Cx3cr1* ^+^ MF, *Folr2* ^+^ MF, *Mki67* ^+^ MF, and *S100a9* ^+^ MF (Figure 5A). Feature plots illustrated the marker expression patterns (Figure 5B), while comparative analyses identified subtype‐specific DEGs, with detailed signatures presented in Figure S4A.

A temporal expression heatmap depicted representative marker genes for each macrophage subtype across five time points (Figure 5C). Integrated analysis combining GO enrichment with longitudinal expression trends revealed distinct functional programs characterizing each subset (Figure 5D), indicating temporally regulated functional specialization.

Consistent with prior findings (Lyras et al. 2022), *Folr2* ^+^ MF and *Cx3cr1* ^+^ MF were terminally differentiated subsets in adult tongue. In our data, *Folr2* ^+^ MF, with selected marker genes *Folr2*, *Lyve1*, *Ccl24*, *Fcna*, and *Ednrb*, reached peak expression during the M3 phase, with associated GO terms including complement activation, positive regulation of epithelial cell proliferation, and macrophage cytokine production. *Cx3cr1* ^+^ MF, characterized by *Cx3cr1*, *Mmp12*, *Il1a*, *Ccl3*, and *Tgfbr1*, fluctuated synchronously and was associated with multicellular organismal processes, reproductive regulation, and connective tissue replacement.

Notably, pro‐inflammatory *S100a9* ^+^ MF, which were scarce in young tissue but significantly increased with aging, showed strong enrichment for interleukin‐6 regulation, innate immunity, and NF‐κB signaling pathways. In contrast, *Bgn* ^+^ MF and *Cd209a* ^+^ MF were consistent with persistent sentinel cells, maintaining stable population dynamics throughout lifespan due to their slow turnover and local microenvironment regulation. Furthermore, *Cd207* ^+^ MF showed low expression in neonates, suggesting requiring postnatal migration and differentiation of bone marrow‐derived precursors to establish stable adult populations. Tissue‐resident macrophages underwent proliferative expansion during the neonatal period (evidenced by elevated *Mki67* expression), transitioning to homeostatic maintenance in maturity. These distinct functional profiles revealed subset‐specific activation states. Aligned with the M1/M2 polarization paradigm in aging and inflammation (Li et al. 2023), we assessed polarization signatures across macrophage subtypes at M24 stage. *Folr2* ^+^ MF exhibited a strong M2‐like phenotype, while *S100a9* ^+^ MF showed a dominant M1‐like signature (Figure 5E). These findings supported a mechanistic model that M2‐polarized *Folr2* ^+^ MF maintain taste bud homeostasis in young mice through anti‐inflammatory and tissue‐protective functions. With aging, the balance shifts, showing that pro‐inflammatory *S100a9* ^+^ MF (M1‐like) accumulates while protective *Folr2* ^+^ MF decline, collectively driving age‐related taste dysfunction. These results corroborated previous findings demonstrating that systemic LPS‐induced inflammation similarly reduces *Folr2* ^+^ MF populations in murine tongue (Lyras et al. 2022), suggesting a conserved mechanism of inflammatory‐mediated taste impairment. To investigate age‐related changes in M2‐type macrophages within taste tissues, we performed immunostaining for Arg1 and Cd206, two well‐known markers of M2‐polarized macrophages (Zhang et al. 2024), on CV tissue sections. The number of Arg1^+^ and Cd206^+^ cells was significantly lower in aged mice compared to young mice (Figure 5G,H and Figure S4B,C). Additionally, *Arg1* expression in MF (Figure 5I) and taste cells (MTC, TPC) (Figure 5J) was higher in M3 aged tissue. This age‐related reduction in M2‐polarized MF may reflect a shift in the local microenvironment from an immunoregulatory toward a pro‐inflammatory state in aged taste epithelium.

To further examine the effects of distinct macrophage subtypes on taste cell proliferation and differentiation, we employed an indirect co‐cultured system in which CV‐derived taste organoids were treated with conditioned medium collected from RAW264.7 macrophage polarized into distinct subtypes: M1‐like (LPS + IFNγ) and M2‐like (IL‐4 + IL‐13) (Figure 5K). Conditioned medium from M1‐MF inhibited the growth of taste organoids, markedly reducing organoid size alongside the numbers of *Krt8* ^+^ mature and *Car4* ^+^ Type III taste cells. RT‐qPCR analysis further showed that M1‐MF broadly downregulated key markers across all major taste cell lineages, including Type I (*Entpd2*), Type II (*Plcβ2* and *Gnat3*), and Type III (*Car4*), as well as stemness (*Lgr5*) and proliferation (*Mki67*) markers. In contrast, conditioned medium from M2‐MF increased organoid size and specifically elevated the number of *Krt8* ^+^ mature taste cells compared to normal control and M0‐MF groups. RT‐qPCR analysis confirmed that M2‐MF upregulated *Mki67* and *Gnat3*, while maintaining the expression of *Entpd2*, *Plcβ2*, and *Car4* (Figure 5L–N and Figure S4F–H).

Collectively, these co‐culture results validated that distinct macrophage subsets exert opposing effects on taste cells: M1‐MF broadly inhibit taste organoid maintenance and differentiation, whereas M2‐MF predominantly support stem cell proliferation and lineage homeostasis. The impaired taste cell function observed in aged tissues is therefore likely associated with a shift toward the M1 phenotype. These findings indicate that the balance of macrophage polarization critically determines whether taste tissue undergoes degeneration or regeneration, with the age‐associated accumulation of M1‐like *S100a9* ^+^ MF potentially contributing to impaired taste cell renewal and providing a mechanistic link to inflammation‐related taste dysfunction.

### 2.6 Thbs1 Suppresses Taste Organoid Proliferation and Differentiation

To explore the mechanistic crosstalk between macrophages and taste‐related cells at the aged stage (M24), CellChat analysis revealed a marked up‐regulation of the MIF signaling axis, a pleiotropic pro‐inflammatory pathway linked to chronic low‐grade inflammation in aged tissues (Baechle et al. 2023; Wu et al. 2024). The Mif‐Cd74/Cd44 ligand‐receptor pair emerged as the most prominent interaction between taste and MF subsets, underscoring an age‐associated amplification of immune signaling in MTC and TPC (Figure 6A and Figure S5A). Immunostaining of CV sections confirmed increased numbers of Cd74^+^ and Mif^+^ cells in aged CV tissue (Figure 6B,C and Figure S4D,E), with elevated *Cd74* expression in MF at M24 (Figure 6D).

Notably, nearly all taste cell populations actively sent chemotactic signals to recruit macrophages, except for *S100a9* ^+^ MF (Figure 6A), suggesting that these cells accumulated during aging primarily through peripheral infiltration or local expansion rather than via taste‐tissue‐derived chemotactic cues, thereby reinforcing their pathogenic rather than homeostatic role. Intercellular interaction analysis further revealed that *S100a9* ^+^ MF exhibit the strongest connectivity with all other macrophage subtypes, reflecting a signal‐amplification and positive‐feedback recruitment mechanism within the inflammatory niche (Figure S5B). Delving deeper into ligand‐receptor pairs, we found that *S100a9* ^+^ MF engaged TPC via the Thbs1‐Sdc axis to suppress TPC proliferation, whereas *Cx3cr1* ^+^ and *Folr2* ^+^ MF communicated with Type II/III MTC through the Grn‐Sort1 interaction to fine‐tune downstream signaling pathways (Figure 6E). Violin plots illustrated temporal expression of key mediators: *Grn* and *Thbs1* in macrophage subsets, *Sdc1* in TPC, and *Sort1* in MTC across each developmental stage (Figure 6F).

To investigate the role of *Thbs1* in taste cell development, we supplemented taste organoid culture medium with recombinant THBS1 protein. Addition of Thbs1 significantly reduced organoid proliferation, as evidenced by decreased number of Ki67^+^ and Krt8^+^ cells (Figure 6G,H). We further confirmed that THBS1 treatment downregulated the mRNA expression of *Lgr5*, *Plcβ2*, *Krt8*, and *Car4* using our previously established RT‐qPCR protocol (Figure 6K) (Ren et al. 2021). Given that Thbs1 is a known physiological regulator of latent Tgfβ1 activation (Murphy‐Ullrich and Suto 2018; Ribeiro et al. 1999), we next examined the effect of Tgfβ1 alone or in combination with Thbs1. Tgfβ1 alone markedly inhibited organoid growth, and co‐administration of Thbs1 further enhanced this inhibition (Figure 6J). These findings suggest that Thbs1 directly suppresses taste organoid proliferation and also synergizes with Tgfβ1 to enhance this inhibitory effect.

Together, these findings suggested an integrated cell–cell communication network that elucidates the dynamic crosstalk between taste‐related cells and macrophages during inflamm‐aging, providing a framework for targeting these circuits to ameliorate age‐related taste dysfunction.

### 2.7 Age‐Related Gene Regulatory Networks of Taste Cells

To explore transcriptional regulatory networks governing aged taste‐cell fate (M24), we interrogated Thbs1‐ and Grn‐mediated gene regulation in TPC and MTC subsets (Table S6). Nichenet analysis predicted *Hes1*, *Icam1*, *Nfkbia*, *Stat3*, *Egr1/2*, *Hsp90ab1*, *Gadd45b*, and *Ctnnb1* as key Thbs1 targets (Figure 7A). *Egr1* showed subtype‐specific repression in *Lgr5* ^+^, *Mki67* ^+^, and *Krt5* ^+^ TPC but induction in *Shh* ^+^ TPC. *Hes1* and *Egr2* were selectively up‐regulated in *Mki67* ^+^ and *Shh* ^+^ TPC, respectively, while most other targets were down‐regulated. As core transducers of the Wnt/β‐catenin axis, suppression of *Stat3* and *Ctnnb1* in *Shh* ^+^ TPC aligned with age‐related Wnt signaling decline (Gaillard and Barlow 2011). The divergent responses reflect subtype‐dependent regulatory roles of *Thbs1*. *Grn* exhibited a distinct pattern by predominantly upregulating genes in Type II/III MTC, including immediate early genes (*Btg2*, *Fos*, *Junb*, *Jund*), stress response regulators (*Dusp1*, *Txnip*), and immune/epigenetic markers (*H2‐D1*, *H3f3b*), while specifically downregulating pro‐proliferative drivers such as *Stat3* and *Stmn1* in Type II MTC (Figure 7B). Thus, Grn couples cytoprotective and differentiation‐associated signals with repression of growth‐promoting pathways, acting as a safeguard against age‐related functional decline in mature taste cells.

Subsequently, we identified transcription factors (TFs) with significant changes (|Log2FC| > 0.5, *p* < 0.05) at M24. Heatmap revealed that *Hes1*, *Jun*, *Junb*, *Klf4*, *Sox4*, *Hes6, Ovol3* and *Uqcrb*, were highly expressed, particularly in MTC subtypes and *Shh* ^+^ TPC (Figure 7C). Regulatory networks revealed several centrally positioned TFs as potential master regulators (Figure 7D). Cell‐type‐specific analysis showed that *Srf*, *Junb*, and *Jun* were upregulated in Type III MTC; *Bmyc* and *Etv1* in Type II MTC; and *Jund*, *Egr1*, and *Atf3* in Type I MTC and Shh^+^ TPC (Figure 7E and Table S7).

Using hdWGCNA, we constructed gene co‐expression networks in TPC and MTC during aging. A dendrogram (Figure S6A) identified 17 modules (Figure S6B). Modules 1, 8, and 16 were selected for their MTC/TPC‐specific and pan‐expression patterns (Figure 7F). For each, we constructed gene co‐expression networks showing the top 15 hub genes (central) with 20 peripheral genes (nodes represent individual genes and edges depict co‐expression relationships). Module 1 (MTC‐specific) hub genes included taste receptors (*Pkd1l3*, *Pkd2l1*, Snap25) and neurodegeneration‐associated genes (*Ddc*, *Gch1*, *Uchl1*) (Buneeva and Medvedev 2024; Pan et al. 2020; Pereira et al. 2023), suggesting links between taste aging and neurodegeneration.

Our previous study demonstrated a decline in the unfolded protein response (UPR) in aged TPC compared to adult TPC (Ren et al. 2024), indicative of reduced protein synthesis capacity. Notably, Module 8 (TPC‐specific) hub genes were enriched for ribosomal genes, implying compensatory increase in ribosome biogenesis to counteract age‐related functional decline. However, given mitochondrial dysfunction and energy metabolism in senescent cells, this compensatory may ultimately contribute to senescence or exhaustion. Module 16 (pan‐expression) hub genes co‐expressed inflammation‐related genes (*Gbp2*, *Tpst1*, *Traf6*, *Il3ra*) (Dickinson et al. 2023; Flayer et al. 2024; Matsumoto et al. 2018; Wang et al. 2022), aging related genes (*Rad9a*, *Rbbp5*, *Senp8*, *Clybl*) (Li et al. 2014; Lieberman et al. 2017), and dual‐function genes (*Dusp7*, *Itga10*) (Luo et al. 2021; Sun et al. 2021). Together, these findings delineated dynamic co‐expression networks in taste cell aging, highlighting potential mechanisms linking ribosomal stress, mitochondrial dysfunction, inflammation, and cell cycle regulation.

## 3 Discussion

Age‐related taste dysfunction impairs nutrition and quality of life in the elderly, but remains poorly understood. Our study provides the first comprehensive spatiotemporal atlas of taste bud aging by integrating single‐cell and spatial transcriptomics across five developmental stages. We reveal that age‐related taste decline is driven by two interconnected mechanisms: progressive depletion of stem/progenitor cell regenerative capacity and establishment of a pro‐inflammatory niche through macrophage phenotypic switching. Stem cell exhaustion is a hallmark of aging (López‐Otín et al. 2023). Adult stem cells exhibit age‐related functional decline, reflected by reduced cell number and dysregulated proliferation (Easwaran et al. 2022). Our study extends these findings to the gustatory system, demonstrating a significant age‐dependent reduction in TPC populations, especially *Lgr5* ^+^ TPC, which correlated with diminished taste sensitivity in aged mice. In vitro taste organoid experiments confirmed impaired regenerative potential of aged TPC. Furthermore, we uncovered marked downregulation of Wnt/β‐catenin signaling and sensory epithelium development pathways, both essential for stem cell maintenance and differentiation (Nusse and Clevers 2017). This age‐related stem cell depletion may result from loss of self‐renewal capacity, apoptosis, or senescence, though the precise mechanisms governing stem cell fate decisions remain incompletely understood.

Our study further identified inflamm‐aging as a central mechanism underlying taste dysfunction. Given the crucial role of macrophages in tongue development and immune defense (Lyras et al. 2022), we observed a significant imbalance between resident and pro‐inflammatory macrophages in aged tongue tissues. *S100a9* ^+^ MF emerged as key mediators, displaying age‐dependent accumulation and progressive pro‐inflammatory polarization. Intriguingly, neonatal mice exhibited a transient *S100a9* ^+^ MF spike at P0, likely representing a developmentally programmed, acute inflammatory response to postnatal food‐ and microbe‐derived antigens, facilitating taste bud maturation and mucosal barrier formation (Wang, Wang, Liu, et al. 2025; Xia et al. 2017). This contrasts sharply with the chronic pro‐inflammatory state of aging.

Recent evidence identifies mouse Type I taste bud cells as intrinsic immune‐competent cells that share key phenotypic and functional features with macrophages, including the expression of F4/80, CD11b, CD11c, CD64, and the ability to respond to inflammatory stimuli. In particular, Type I cells can be polarized toward an M2‐like state upon IL‐4 stimulation, as evidenced by increased arginase 1 (Arg1) expression (Hichami et al. 2023). Additionally, TNFα^+^ cells reside in taste buds under healthy conditions (Feng et al. 2012), suggesting a protective role against bacterial stimulation.

Age mice showed reduced *Arg1* ^+^/*Cd206* ^+^ M2‐like cells in CV compared to young controls. Given that Type I cells express *Arg1* under M2‐polarizing conditions, aging may impair their adoption of a pro‐regenerative M2‐like phenotype, reflecting a shift from an immunoregulatory to a pro‐inflammatory state. This interpretation is further supported by our organoid co‐culture experiments, where M1‐conditioned medium broadly inhibited organoid growth and taste lineage differentiation, whereas M2‐conditioned medium promoted organoid expansion and selectively supported Type II (*Gnat3*) taste cell differentiation.

Collectively, our findings suggest that an age‐related loss of M2‐like polarization—potentially intrinsic to Type I taste cells or within their immune niche—may compromise taste tissue maintenance and regeneration. Future therapeutic strategies aimed at restoring M2‐like polarization in Type I cells or the local macrophage niche may represent a promising target to ameliorate age‐related taste dysfunction.

Nevertheless, two methodological considerations in our approach warrant attention. First, supplementing the standard, serum‐free taste organoid medium with 50% MF‐conditioned medium (prepared in DMEM containing 10% FBS) introduced a final FBS concentration of approximately 5%. While this serum component may have contributed to baseline organoid size expansion and *Mki67* expression observed under M0 and M2 conditions, M1‐conditioned medium still markedly suppressed organoid expansion despite these pro‐growth serum conditions, highlighting the overriding inhibitory potency of M1‐derived factors. Second, given that *Krt8* is not restricted to taste cells but is also expressed in von Ebner's gland ductal and acinar epithelial structures, which may be retained as minor non‐taste populations in lingual organoid cultures (Verweij et al. 2025), we deliberately integrated Krt8 profiling with lineage‐specific RT‐qPCR measurements of Type I, Type II, and Type III taste cell markers. This multi‐marker strategy ensured a rigorous, unambiguous evaluation of taste‐specific cell responses.

Cell–cell communication analysis further elucidated the molecular mechanisms bridging macrophage activation states to progenitor dysfunction, identifying *S100a9* ^+^ MF as the primary source interacting with TPC subtypes via the Thbs1‐Sdc axis. As a key matricellular protein, THBS1 drives fibrotic progression through dual activation of TGF‐β and NF‐κB signaling, which stimulate excessive collagen deposition and promote chronic low‐grade inflammation via elevated IL‐6 and TNF‐α secretion (Haga et al. 2024; Vanhoutte et al. 2024). Age‐related *Thbs1* upregulation suggests its role in inflamm‐aging and extracellular matrix remodeling. Additionally, upregulation of *Hes1* (a Notch pathway target gene) and *Egr2* (a neurodevelopmental transcription factor) suggests that *Thbs1* may partially sustain progenitor cell activity via Notch or neurodevelopmental pathways, though insufficient to counteract the overall functional decline. Furthermore, Grn emerged as a critical regulator of taste cell homeostasis, with its age‐related decline driving sensory impairment through reduced stress‐responsive gene activation, disrupted immune surveillance, and imbalanced differentiation‐proliferation equilibrium.

Crucially, through taste organoid experiments, we demonstrated that recombinant THBS1 faithfully phenocopied the key inhibitory features observed with M1‐MF conditioned medium, specifically suppressing organoid expansion and downregulating both stemness (*Lgr5*) and taste cell markers (*Plcβ2*, *Krt8*, *Car4*). This inhibitory effect was further enhanced by Tgfβ1 co‐treatment, aligning with the established role of Thbs1 as a physiological activator of latent Tgf‐β (Ribeiro et al. 1999; Murphy‐Ullrich and Suto 2018). Exogenous Thbs1 may activate endogenous latent Tgf‐β within the organoid microenvironment, amplifying Tgf‐β signaling beyond exogenous active Tgf‐β alone.

Thbs1 reduced differentiated taste cells and altered organoid size, suggesting it modulates cell fate rather than generally inhibiting proliferation. Thbs1 may divert progenitor cells away from the taste cell lineage, relevant to aging where taste stem cells already exhibit impaired differentiation.

In our previous study, we identified stem/progenitor cell exhaustion in taste progenitor cells as a key driver of age‐related taste decline from a cellular atlas perspective. Here, we further demonstrate that inflammatory microenvironment remodeling during inflamm‐aging, particularly macrophage phenotypic switching, drives the reduced differentiation capacity of taste stem cells, with macrophage‐derived Thbs1‐mediated suppression representing a critical step in this process.

One limitation of our study is the underrepresentation of taste epithelial cells in the anterior FF dataset due to the need to expand microdissection areas. Sensitivity analyses confirmed that our key conclusions are robust and primarily driven by posterior tongue data. Therefore, while our findings provide a comprehensive atlas of age‐related taste decline, conclusions regarding taste cell lineage dynamics are weighted toward the posterior taste fields (CV and FL), and anterior FF regulation will require dedicated future studies.

In conclusion, our study provides a comprehensive understanding of age‐related taste dysfunction, clarifying the roles of progenitor cell depletion and inflammatory niche remodeling while identifying promising therapeutic targets, including macrophage polarization and the Thbs1‐Sdc axis. This work deepens our understanding of taste dysfunction pathogenesis in the elderly and paves the way for effective therapeutic interventions to improve their quality of life.

## 4 Methods and Materials

### 4.1 Single‐Cell RNA Sequencing (scRNA‐Seq)

Wild‐type C57BL/6J mice at P0, P14, M3, M12, and M24 were obtained from Shanghai Bikai Laboratory Animal Company Ltd. (Shanghai, China). The animal handling procedures were approved by the Ethics Committee of Laboratory Animals in Naval Medical University (Permit Number: SYXK2022‐0011). Tissue samples were collected from CV, FL, and FF papillae. For each sample, six tongue pieces from different mice were pooled to minimize biological variability. Single‐cell suspensions were obtained by enzymatic digestion with 0.25% trypsin–EDTA (37°C, 20 min), followed by centrifugation (1000 rpm, 5 min). Cells were resuspended in HBSS buffer and filtered through a 40‐μm cell strainer to ensure a high‐quality single‐cell suspension.

### 4.2 Library Construction and Sequencing

cDNA libraries were prepared using the DNBelab C4 platform following manufacturer protocols. Single‐stranded DNA (ssDNA) library quality was assessed using the Qubit ssDNA Assay Kit. Sequencing libraries were then converted into DNA nanoballs (DNBs) and sequenced on the MGI DNBSEQ‐T10 platform.

### 4.3 Processing of scRNA‐Seq Data

Raw sequencing data were processed using MGI's dnbc4tools pipeline (https://github.com/MGI‐tech‐bioinformatics/DNBelab_C_Series_HT_scRNA‐analysis‐software), which performed alignment of FASTQ files to the mouse reference genome (GRCm39) to generate the initial gene expression matrix. Following UMI‐based filtering to remove potential PCR duplicates and low‐quality data, the processed expression matrix was obtained for subsequent analytical steps.

### 4.4 scRNA‐Seq Data Integration and Cell‐Type Identification

scRNA‐seq data were analyzed using Seurat (version 4.4.0), integrating newly generated and published datasets (Ren et al. 2024), encompassing CV and FL papillae from M3, and M24 mice. Following uniform quality control criteria retaining cells with > 500 detected genes, > 800 UMIs, and < 10% mitochondrial gene content, the analytical pipeline included normalization and scaling of gene expression data, identification of integration anchors using the “FindIntegrationAnchors” function, and dataset integration via the “IntegrateData” function. Subsequent dimensional reduction and clustering analysis (resolution parameter = 0.5) enabled cell population identification, with each cluster annotated based on expression of at least two established cell type‐specific marker genes.

### 4.5 Stereo‐Seq Spatial Transcriptomics Analysis

Taste papillae (CV, FL, and FF) from P14, M3, M12, and M24 mice were embedded in pre‐cooled OCT compound, flash‐frozen on dry ice, and stored at −80°C. Using a Leica CM1950 cryostat, 10 μm transverse sections were mounted on Stereo‐seq chips. Tissue sections underwent sequential processing including: 0.1× SSC buffer (Thermo AM9770) washes with RNase inhibitor (NEB M0314L, 0.05 U/μL), pepsin‐based permeabilization (0.1% in 0.01 M HCl, pH 2.0, 37°C for 18 min), and mRNA capture using DNB probes. Reverse transcription was performed at 42°C overnight, followed by RT product digestion (37°C, 30 min) and PCR amplification. Purified products were used for DNB library construction and sequenced on an MGI DNBSEQ‐T10 platform, generating subcellular‐resolution spatial gene expression matrices for downstream analysis.

### 4.6 Stereo‐Seq Data Processing Pipeline

Raw sequencing data were computationally processed through the SAW analysis pipeline (Gong et al. 2024), where PCR‐amplified duplicates of molecular identifiers (MIDs) were computationally removed using the handleBam algorithm (https://github.com/BGIResearch/handleBam) to generate a high‐resolution coordinate identifier expression matrix. Manual image registration aligned spatial expression profiles with ssDNA staining patterns. CellProfiler was used for cellular segmentation to precisely delineate individual cell boundaries from registered ssDNA images. MIDs were aggregated and summed on a per‐gene basis to construct a comprehensive single‐cell gene expression matrix. Finally, leveraging anatomical landmarks and histological features, taste bud‐specific regions were computationally identified and extracted from the ssDNA images, followed by coordinate‐based filtering of cells and their associated gene expression profiles for subsequent analytical procedures.

### 4.7 Integrated Analysis of Stereo‐Seq and scRNA‐Seq Data for Cell‐Type Annotation

Cell‐type annotation of spatial transcriptomic data was achieved through integrative analysis with matched scRNA‐seq reference datasets. For each taste papilla type, we performed data integration using Seurat's “FindIntegrationAnchors” and “IntegrateData” functions to harmonize the spatial transcriptomic profiles with their corresponding pre‐annotated scRNA‐seq datasets. Quality assessment revealed minimal batch effects in all integrated datasets, as evidenced by the absence of data type‐specific clustering patterns. Cell‐type identities were then assigned to each cluster based on the predominant cell population identified in the matched scRNA‐seq reference data, with this annotation subsequently applied to all spatially resolved cells within the corresponding cluster, thereby enabling comprehensive cell‐type mapping across both molecular and spatial dimensions.

### 4.8 Gene Ontology (GO) Enrichment Analysis

Functional annotation of DEGs was performed using the clusterProfiler package (version 4.6.2) in R, with specific focus on biological processes (“ont = BP”) at a significance threshold of *p* < 0.05 (“*p*valueCutoff = 0.05”) through the “enrichGO” function. Resultant enrichment profiles were visualized using ggplot2 (version 3.5.1) to illustrate significantly enriched GO terms and their relationships to the identified DEGs.

### 4.9 Identification and Analysis of Age‐Related DEGs

Age‐associated transcriptional changes were systematically identified by performing pairwise comparisons between the M24 timepoint and each of the other four developmental stages (P0, P14, M3, M12) for every cell population using Seurat's “FindMarkers” function. Stringent statistical thresholds were applied to define age‐related DEGs, requiring both statistical significance (adjusted *p*‐value < 0.05) and meaningful biological effect size (absolute average log2 fold‐change > 0.25). For subsequent functional characterization of TPC and MTC, we implemented more conservative criteria (avg_log2FC > 0.5, *p*_val_adj < 0.05) to focus on the most robust age‐associated changes. Comparative analysis of DEG sets across timepoints was visualized using ggVennDiagram (version 1.2.3) to elucidate temporal patterns of transcriptional regulation during aging.

### 4.10 Gene Set Variation Analysis (GSVA) of Biological Processes Across Developmental Stages

Gene Set Variation Analysis was performed using GSVA (version 1.46.0) to quantify activity changes in Gene Ontology biological processes (MsigDB M5 collection) across developmental timepoints. This single‐sample enrichment approach generated continuous enrichment scores for each GO term, enabling systematic evaluation of potential functional alterations during taste cell maturation and aging. Resultant activity profiles were visualized as a clustered heatmap using pheatmap (version 1.0.12), with hierarchical clustering revealing stage‐specific patterns of biological process regulation.

### 4.11 Pseudotemporal Analysis of Differentially Expressed Genes in Taste Cell Lineages

We employed Monocle2 (version 2.22.0) to identify pseudotime‐dependent transcriptional changes in TPC and MTC across five developmental timepoints. The analysis pipeline consisted of: initial detection of temporally regulated genes using the “differentialGeneTest” function (*q*‐value < 0.05); pseudotemporal ordering of cells based on these candidate genes; and visualization of expression dynamics through “plot_pseudotime_heatmap”. To ensure robust detection of lineage‐specific patterns, we applied distinct filtering criteria for each cell population—requiring expression in > 3500 cells (*p* < 0.0001) for TPC and > 1000 cells (*p* < 0.0001) for MTC, reflecting their differing cellular abundances while maintaining consistent statistical stringency.

### 4.12 Pseudotemporal Trajectory Analysis of Taste Cell Development and Aging

Developmental and aging trajectories of taste cells were reconstructed using Slingshot (version 2.12.0), with Krt5^+^ TPC designated as the root state for all pseudotemporal analyses across five developmental timepoints. The “slingshot” function was employed to computationally infer cellular progression paths, followed by lineage tracing visualization through integration of the “getLineages” function with UMAP dimensional reduction, enabling comprehensive mapping of potential differentiation routes and age‐related transitions within the taste cell populations.

### 4.13 Single‐Cell Pathway Activity Scoring and Analysis

Pathway activity dynamics were quantified at single‐cell resolution using Seurat's “AddModuleScore” function. Curated gene sets obtained from the Molecular Signatures Database (MSigDB, https://www.gsea‐msigdb.org/gsea/msigdb) were first filtered to include only genes detected in our expression matrix. Single‐cell pathway scores were then computed based on normalized gene expression rankings, generating ModuleScores that were subsequently analyzed across developmental timepoints and cell types. The distributions of these scores were visualized through violin and box plots using ggplot2, while statistical comparisons between adjacent developmental stages were performed with ggpubr's “stat_compare_means” function (version 0.6.4) to identify significant alterations in pathway activities during taste cell maturation and aging.

### 4.14 Identification and Characterization of Age‐Associated Inflammatory Genes

The inflammatory gene signature in M24 taste tissues was systematically analyzed by intersecting age‐related DEGs (*p*_adj < 0.05, |log2FC| > 0.25) with the canonical inflammatory response gene set (M5 collection) from MSigDB. Quantitative analysis of inflammatory DEG prevalence across cell types and comparison groups was visualized through clustered heatmaps, while temporal expression patterns of key inflammatory mediators were profiled using longitudinal heatmap visualization to delineate their dynamic regulation throughout the aging process. This multi‐faceted approach enabled comprehensive evaluation of both the scope and progression of inflammatory activation in aged taste tissues.

### 4.15 Spatial co‐Localization Analysis of Immune‐Taste Cell Interactions

Spatial co‐occurrence patterns between immune cells and taste cell populations in CV were quantitatively analyzed using squidpy (version 1.6.1) with a defined interaction distance threshold of 100 μm. The “gr.co_occurrence” function was applied to raw spatial coordinates to compute: conditional probability [*p*(exp|cond)] of observing immune cells (exp) within the microenvironment of taste cell clusters (cond), and baseline probability [*p*(exp)] of immune cell occurrence within the specified radius. Resultant co‐localization profiles were visualized via “pl.co_occurrence”, enabling statistical evaluation of spatially restricted immune‐taste cell interactions in aged taste bud microenvironments.

### 4.16 Cell–Cell Communication Network Analysis in Taste Tissue Aging

Cell–cell communication networks between immune cells and taste cells were systematically analyzed using CellChat (version 1.4.0). Communication networks for TPC, MTC, TC, and MF at M3 and M24 were constructed from normalized scRNA‐seq data and temporally integrated with “mergeCellChat” function. Subtype‐resolution analysis of MF and taste cells at M24 identified Thbs1 (from S100a9^+^ MF) and Grn (from Cx3cr1^+^/Folr2^+^ MF) as key age‐associated ligands. To elucidate regulatory roles, DEGs in target taste subtypes (M24 vs. other timepoints) were identified using Seurat's Wilcoxon test, and ligand‐target interactions were predicted by NicheNet (version 2.2.0) via “prepare_ligand_target_visualization” function, integrating DEGs with its prior‐knowledge database to map age‐related changes in immune‐taste cell communication networks.

### 4.17 Transcriptional Regulatory Network Analysis in Taste Cell Aging

We employed SCENIC (version 0.11.2) to reconstruct transcriptional regulatory networks in TPC and MTC during aging, using mm10 transcription factors (TFs) as input for GRNboost network inference, RcisTarget motif analysis, and AUCell regulon scoring. Age‐associated regulatory changes were identified by intersecting network TFs with DEGs from M24 vs. other time points (|avg_log2FC| > 0.5, *p*_adj < 0.05). For each taste cell subtype, age‐related DEGs were separately derived to refine cell‐type‐specific TFs and classify them as upregulated or downregulated during aging. The resulting TF modules and regulatory relationships were visualized as interactive networks in Cytoscape (version 3.7.2), highlighting key transcriptional drivers of taste cell maturation and age‐related functional decline.

### 4.18 High‐Dimensional co‐Expression Network Analysis of Aged Taste Cells

To investigate coordinated gene expression in aged taste cells, we performed hdWGCNA (version 0.3.1). Metacell was constructed and normalized using “MetacellsByGroups” and “NormalizeMetacells” functions to minimize technical variability while retaining biological signals. Consensus co‐expression networks were built with “ConstructNetwork” incorporating soft‐thresholding and topological overlap matrix calculations to establish reliable gene–gene relationships. Network modules were characterized via “ModuleEigengenes” and “ModuleConnectivity” functions to identify central network hubs and connectivity patterns, uncovering potential regulatory mechanisms involved in taste cell maintenance within aging microenvironments.

### 4.19 Immunofluorescence

For tissue immunostaining, mice were sacrificed under deep anesthesia with intraperitoneal injection of ketamine‐xylazine (200 and 15 mg/kg body weight). The CV, FL, and FF tissues were dissected from tongues and fixed in 4% paraformaldehyde (Sigma Aldrich), infiltrated in 30% sucrose, and embedded in OCT. Sections (20 μm) were cut on a cryostat (Leica CM1950).

For organoid staining, cultured taste organoids were harvested and fixed in 4% paraformaldehyde for 30 min at 4°C. Sections or organoids were blocked in PBS containing 0.3% Triton X‐100 and 5% BSA for 1 h, then incubated with primary antibodies overnight at 4°C, followed by secondary antibodies for 1.5 h at room temperature. The primary and secondary antibodies used were listed in Table S8A,B. For all immunostaining, DAPI was employed to counterstain the cell nuclei. Fluorescent images were captured under a SP8/Leica confocal microscope equipped with LAS AF Lite software.

### 4.20 Construction and Evaluation of Supervised Classification Machine Learning Model

Supervised machine learning was conducted using the Python package sklearn (version 1.3.2). The data matrix consists of taste cells from five time points and the corresponding top 1000 DEGs for each of the time points. The dataset was randomly divided into training/testing subsets at a 3:2 ratio. The testing dataset was exclusively used for evaluating the trained model and was not exposed to the training phase. The training dataset was modeled using ensemble classification algorithms (Random Forest and Support Vector Machine) with 5‐fold cross‐validation. After training, the test dataset was used to evaluate model performance via Receiver Operating Characteristic curves and confusion matrices.

### 4.21 Construction of 3D Taste Organoids

In accordance with our previous study (Takeda et al. 2013), single cells isolated from the CV papillae of P0, M3, and M24 mice of different ages were cultured in conditioned medium with matrigel added. The culture medium was replaced once every 3 days. Upon completion of 14 days of cultivation, the organoids were collected and processed for immunostaining.

### 4.22 Stimulation of Taste Organoids

On day 3 after taste organoid passage, Thbs1 (MCE, #HY‐P701325) was added to the culture medium at final concentrations of 100, 200, and 300 ng/mL for stimulation. On day 6 following stimulation, bright‐field images of each group were captured, and immunofluorescence staining was performed to validate changes in taste cells.

For macrophage stimulation, on day 3 after taste organoid passage, the culture medium was replaced with a 1:1 mixture of differentiation medium and macrophage culture supernatant for further differentiation. On day 9 of co‐culture, the cultures were examined under a microscope and subjected to immunofluorescence staining.

### 4.23 Collection of Macrophage Supernatant

RAW264.7 cells were cultured in DMEM (high sucrose) supplement with 10% FBS (Zhong Qiao Xin Zhou Biotechnology, Cat No. ZM0098) at 37°C in 5% CO_2_, with medium renewal every other day. Upon reaching 5 × 10^5^ cells/well, cells were stimulated. For M1 group, complete medium was supplemented with LPS (100 ng/mL) and IFNγ (20 ng/mL). For M2 group, complete medium was supplemented with IL‐4 (10 ng/mL) and IL‐13 (10 ng/mL). Then cells were cultured for an additional 48 h. Supernatants containing secreted factors from polarized M1 and M2 macrophages were collected and stored for subsequent co‐culture with taste organoids.

### 4.24 Statistical Analysis

IBM SPSS version 24.0 and Graphpad Prism version 8.0 (Graphpad Software, La Jolla, Calif) were used for the statistical analysis. For normally distributed data, we used the unpaired Student *t*‐test to compare the differences between two groups and the ANOVA with Tukey *post hoc test* to adjust multiple comparisons. For non‐normally distributed data, the two‐tailed Mann–Whitney *U* test was used for comparisons between two groups, and the Kruskal‐wallis test with the Dunn *post hoc test* was employed for multiple comparisons. Correlations were assessed using the Spearman rank correlation. The *p* values were shown as indicated (*p < 0.05, **p < 0.01, ***p < 0.001, ****p < 0.0001).

## Author Contributions

W.R., H.L., H.L., and Y.Y., designed the research; W.Z., Y.Y., W.X., L.L., and M.Z. analyzed the data; X.C., Y.X., and E.W. performed the research; X.L. provided critical advice on manuscript revision; W.R., W.Z., Y.Y., and H.L., wrote the paper.

## Funding

This work was supported by National Natural Science Foundation of China Grants (82271136 to W.R., U23A20439 to H.L., 82571283 and 32271044 to Y.Y.); Science and Technology Commission of Shanghai Municipality (23ZR1409600 to Y.Y.); Shanghai Municipal Education Commission, the Shanghai Eastern Scholar Program (GZ2022006 to Y.Y.); Shenzhen Science and Technology Program (RCJC20221008092804002 to H.L.).

## Conflicts of Interest

The authors declare no conflicts of interest.

## Supporting information


**Figure S1:** Spatially resolved single‐cell transcriptomic atlas of mouse taste papillae.

**Figure S2:** Cell identify marker expression and age associated pathway remodeling across three tissues.

**Figure S3:** Gene expression patterns in taste cell subtypes.

**Figure S4:** Comparative analysis of MF polarization and functional effects between young and aged taste papillae (related to Figure 5).

**Figure S5:** Cell–cell communication analysis between MF and taste cell subtypes at M24.

**Figure S6:** hdWGCNA analysis showing the gene co‐expression network between MTC and TPC in M24.

**Table S1:** Cell number summary across taste papillae, time points, and cell types.

**Table S2:** GO term of taste sense.

**Table S3:** Gene sets for TPC scoring.

**Table S4:** DEGs and GO enrichment between M24 and other timepoints across MTC and TPC.

**Table S5:** Gene list for scoring analysis.

**Table S6:** The target genes predicted by Nichenet.

**Table S7:** DEGs of each taste subtypes between M24 and other time points.

**Table S8:** A Primary antibodies for immunofluorescence.

**Data S1:** acel70674‐sup‐0015‐DataS1.docx.

## Data Availability

All data needed to evaluate the conclusions in the paper are present in the paper and/or the Supporting Information—S1. Raw sequencing data generated in this study were deposited at CNGB Nucleotide Sequence Archive (accession code: CNP0007410). All original codes supporting this study were hosted on GitHub: https://github.com/Zhaowado/Inflammatory‐aging‐taste. Requests for the data should be submitted to the lead contact W. Ren.
