# Supplementary material for: Molecular Mechanisms of Age‐Related Taste Dysfunction Deciphered by Spatiotemporal Transcriptomics
Source: Aging Cell. 2026 Aug 20;25(9):e70674. doi: 10.1111/acel.70674 (PMC13494364; doi:10.1111/acel.70674)
Supplement: Supplementary file 1 [file ACEL-25-e70674-s001.pdf]

A

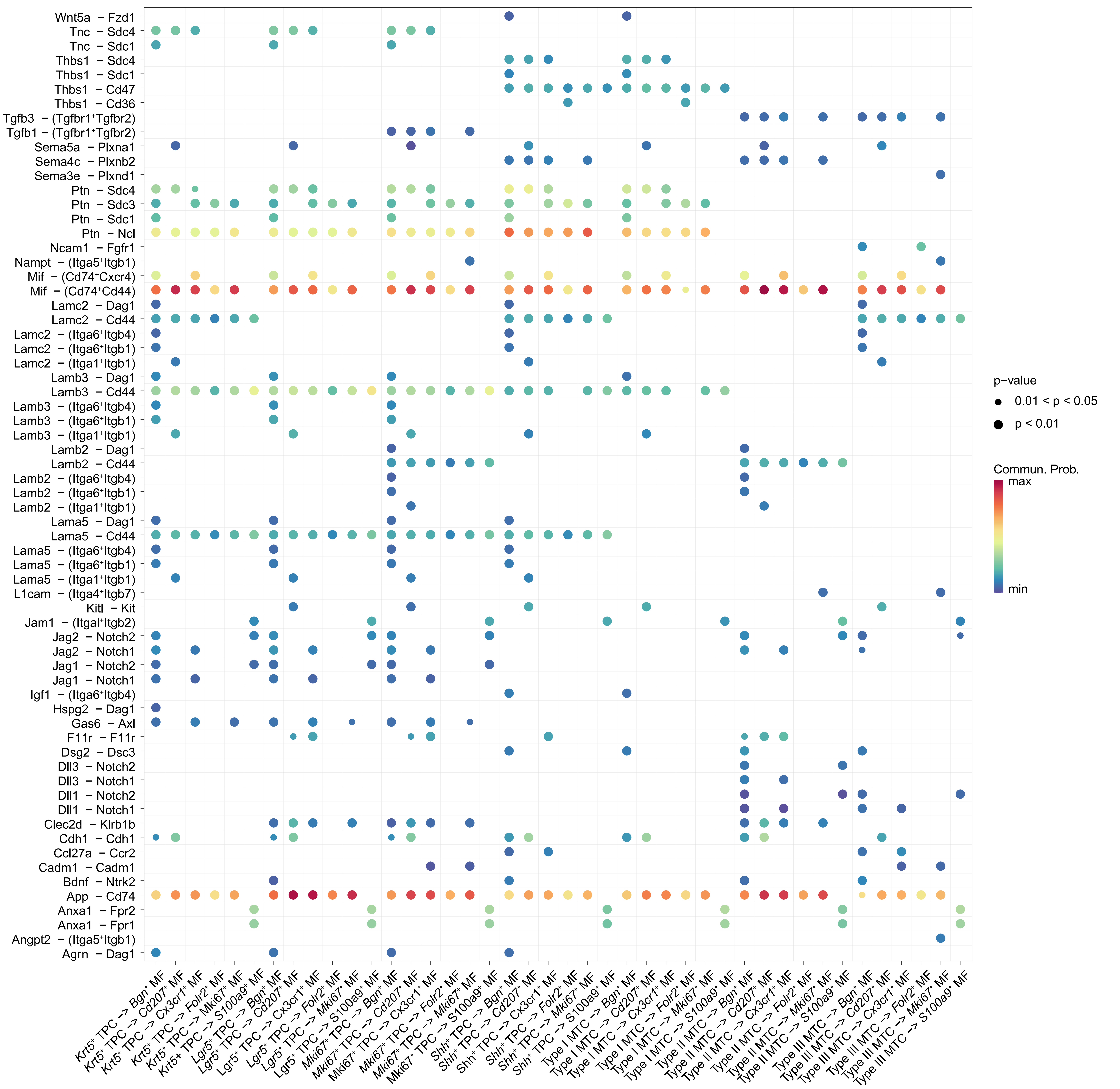

Wnt5a - Fzd1
Tnc - Sdc4
Tnc - Sdc1
Thbs1 - Sdc4
Thbs1 - Sdc1
Thbs1 - Cd47
Thbs1 - Cd36
Tgfb3 - (Tgfbr1+Tgfbr2)
Tgfb1 - (Tgfbr1+Tgfbr2)
Sema5a - Plxna1
Sema4c - Plxnb2
Sema3e - Plxnd1
Ptn - Sdc4
Ptn - Sdc3
Ptn - Sdc1
Ptn - Ncl
Ncam1 - Fgfr1
Nampt - (Itga5+Itgb1)
Mif - (Cd74+Cxcr4)
Mif - (Cd74+Cd44)
Lamc2 - Dag1
Lamc2 - Cd44
Lamc2 - (Itga6+Itgb4)
Lamc2 - (Itga6+Itgb1)
Lamc2 - (Itga1+Itgb1)
Lamb3 - Dag1
Lamb3 - Cd44
Lamb3 - (Itga6+Itgb4)
Lamb3 - (Itga6+Itgb1)
Lamb3 - (Itga1+Itgb1)
Lamb2 - Dag1
Lamb2 - Cd44
Lamb2 - (Itga6+Itgb4)
Lamb2 - (Itga6+Itgb1)
Lamb2 - (Itga1+Itgb1)
Lama5 - Dag1
Lama5 - Cd44
Lama5 - (Itga6+Itgb4)
Lama5 - (Itga6+Itgb1)
Lama5 - (Itga1+Itgb1)
L1cam - (Itga4+Itgb7)
Kitl - Kit
Jam1 - (Itgal+Itgb2)
Jag2 - Notch2
Jag2 - Notch1
Jag1 - Notch2
Jag1 - Notch1
Igf1 - (Itga6+Itgb4)
Hspg2 - Dag1
Gas6 - Axl
F11r - F11r
Dsg2 - Dsc3
Dll3 - Notch2
Dll3 - Notch1
Dll1 - Notch2
Dll1 - Notch1
Clec2d - Klrb1b
Cdh1 - Cdh1
Ccl27a - Ccr2
Cadm1 - Cadm1
Bdnf - Ntrk2
App - Cd74
Anxa1 - Fpr2
Anxa1 - Fpr1
Angpt2 - (Itga5+Itgb1)
Agrn - Dag1
Krt5+ TPC -> Bgn+ MF
Krt5+ TPC -> Cd207+ MF
Krt5+ TPC -> Cx3cr1+ MF
Krt5+ TPC -> Folr2+ MF
Krt5+ TPC -> Mki67+ MF
Krt5+ TPC -> S100a9+ MF
Lgr5+ TPC -> Bgn+ MF
Lgr5+ TPC -> Cd207+ MF
Lgr5+ TPC -> Cx3cr1+ MF
Lgr5+ TPC -> Folr2+ MF
Lgr5+ TPC -> Mki67+ MF
Lgr5+ TPC -> S100a9+ MF
Mki67+ TPC -> Bgn+ MF
Mki67+ TPC -> Cd207+ MF
Mki67+ TPC -> Cx3cr1+ MF
Mki67+ TPC -> Folr2+ MF
Mki67+ TPC -> Mki67+ MF
Mki67+ TPC -> S100a9+ MF
Shh+ TPC -> Bgn+ MF
Shh+ TPC -> Cd207+ MF
Shh+ TPC -> Cx3cr1+ MF
Shh+ TPC -> Folr2+ MF
Shh+ TPC -> Mki67+ MF
Shh+ TPC -> S100a9+ MF
Type I MTC -> Bgn+ MF
Type I MTC -> Cd207+ MF
Type I MTC -> Cx3cr1+ MF
Type I MTC -> Folr2+ MF
Type I MTC -> Mki67+ MF
Type I MTC -> S100a9+ MF
Type II MTC -> Bgn+ MF
Type II MTC -> Cd207+ MF
Type II MTC -> Cx3cr1+ MF
Type II MTC -> Folr2+ MF
Type II MTC -> Mki67+ MF
Type II MTC -> S100a9+ MF
Type III MTC -> Bgn+ MF
Type III MTC -> Cd207+ MF
Type III MTC -> Cx3cr1+ MF
Type III MTC -> Folr2+ MF
Type III MTC -> Mki67+ MF
Type III MTC -> S100a9+ MF
p-value
0.01 < p < 0.05
p < 0.01
Commun. Prob.
max
min

B

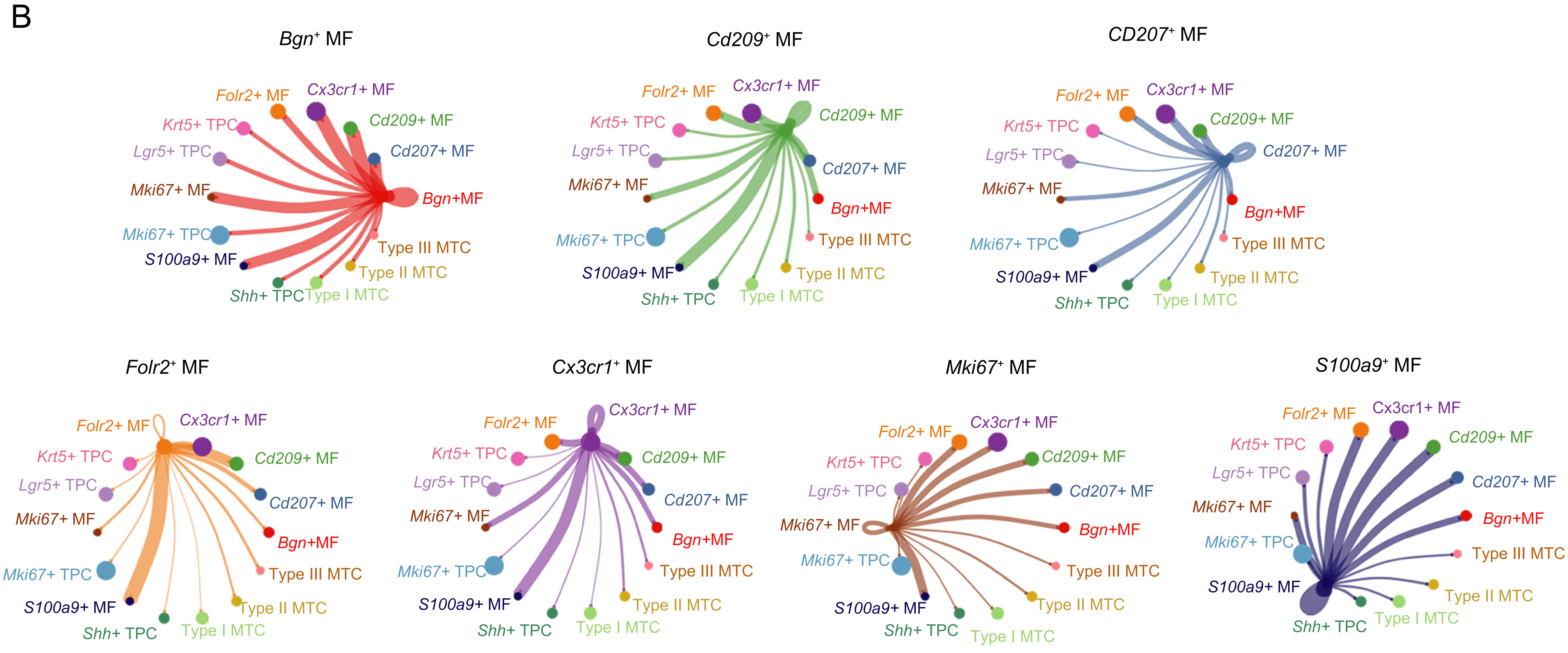

Bgn+ MF
Cd209+ MF
CD207+ MF
Folr2+ MF
Cx3cr1+ MF
Mki67+ MF
S100a9+ MF
Folr2+ MF
Cx3cr1+ MF
Cd209+ MF
Krt5+ TPC
Lgr5+ TPC
Cd207+ MF
Mki67+ MF
Bgn+MF
Mki67+ TPC
S100a9+ MF
Type III MTC
Type II MTC
Shh+ TPC
Type I MTC
