# Supplementary material for: Molecular Mechanisms of Age‐Related Taste Dysfunction Deciphered by Spatiotemporal Transcriptomics
Source: Aging Cell. 2026 Aug 20;25(9):e70674. doi: 10.1111/acel.70674 (PMC13494364; doi:10.1111/acel.70674)
Supplement: Supplementary file 2 [file ACEL-25-e70674-s002.pdf]

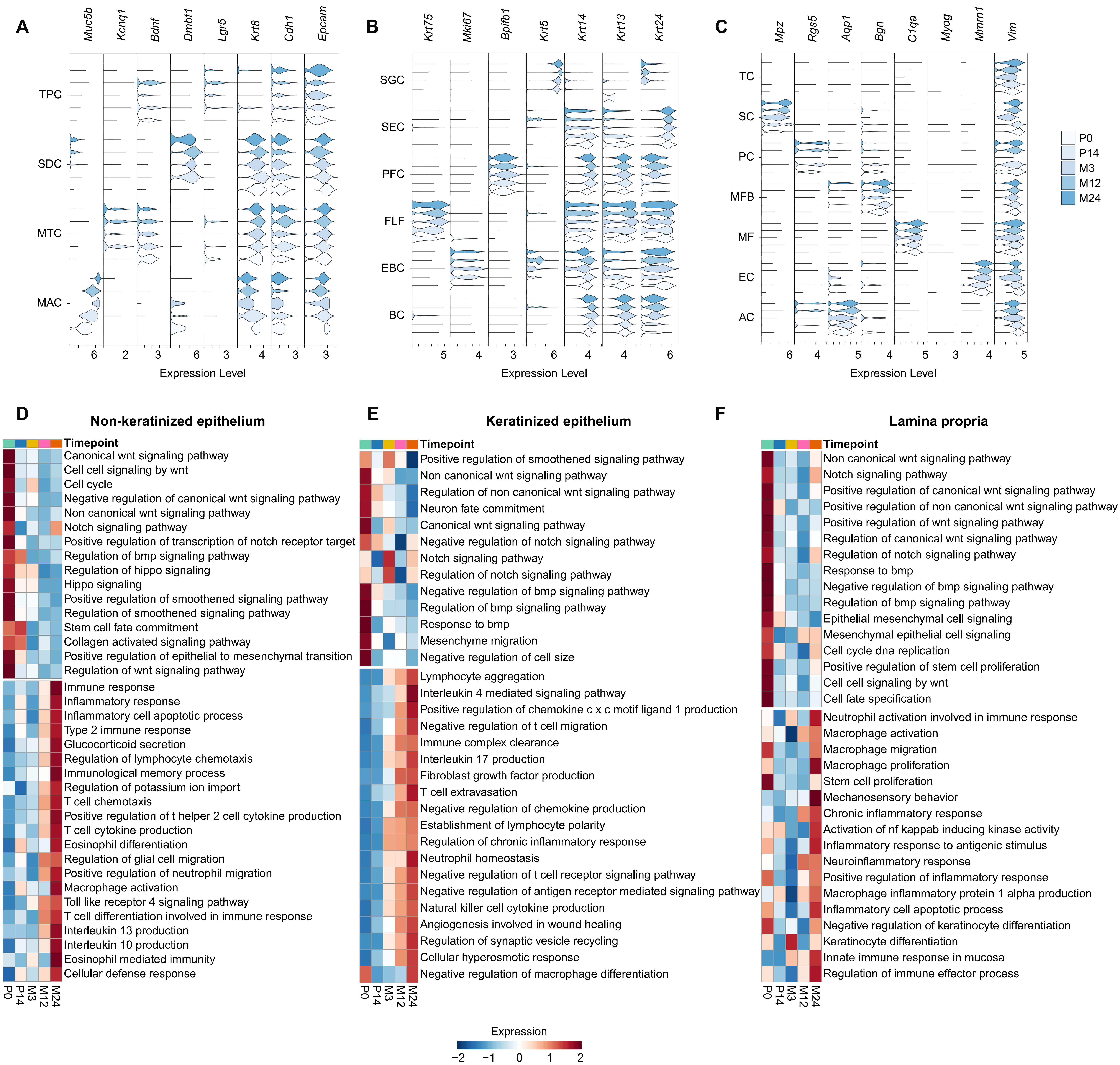
A
Muc5b
Kcnq1
Bdnf
Dmbt1
Lgr5
Krt8
Cdh1
Epcam
TPC
SDC
MTC
MAC
6
2
3
6
3
4
3
3
Expression Level
B
Krt75
Mki67
Bpifb1
Krt5
Krt14
Krt13
Krt24
SGC
SEC
PFC
FLF
EBC
BC
5
4
3
6
4
4
6
Expression Level
C
Mpz
Rgs5
Aqp1
Bgn
C1qa
Myog
Mmrn1
Vim
TC
SC
PC
MFB
MF
EC
AC
6
4
5
4
5
3
4
5
Expression Level
P0
P14
M3
M12
M24
D
Non-keratinized epithelium
Timepoint
Canonical wnt signaling pathway
Cell cell signaling by wnt
Cell cycle
Negative regulation of canonical wnt signaling pathway
Non canonical wnt signaling pathway
Notch signaling pathway
Positive regulation of transcription of notch receptor target
Regulation of bmp signaling pathway
Regulation of hippo signaling
Hippo signaling
Positive regulation of smoothened signaling pathway
Regulation of smoothened signaling pathway
Stem cell fate commitment
Collagen activated signaling pathway
Positive regulation of epithelial to mesenchymal transition
Regulation of wnt signaling pathway
Immune response
Inflammatory response
Inflammatory cell apoptotic process
Type 2 immune response
Glucocorticoid secretion
Regulation of lymphocyte chemotaxis
Immunological memory process
Regulation of potassium ion import
T cell chemotaxis
Positive regulation of t helper 2 cell cytokine production
T cell cytokine production
Eosinophil differentiation
Regulation of glial cell migration
Positive regulation of neutrophil migration
Macrophage activation
Toll like receptor 4 signaling pathway
T cell differentiation involved in immune response
Interleukin 13 production
Interleukin 10 production
Eosinophil mediated immunity
Cellular defense response
P0
P14
M3
M12
M24
E
Keratinized epithelium
Timepoint
Positive regulation of smoothened signaling pathway
Non canonical wnt signaling pathway
Regulation of non canonical wnt signaling pathway
Neuron fate commitment
Canonical wnt signaling pathway
Negative regulation of notch signaling pathway
Notch signaling pathway
Regulation of notch signaling pathway
Negative regulation of bmp signaling pathway
Regulation of bmp signaling pathway
Response to bmp
Mesenchyme migration
Negative regulation of cell size
Lymphocyte aggregation
Interleukin 4 mediated signaling pathway
Positive regulation of chemokine c x c motif ligand 1 production
Negative regulation of t cell migration
Immune complex clearance
Interleukin 17 production
Fibroblast growth factor production
T cell extravasation
Negative regulation of chemokine production
Establishment of lymphocyte polarity
Regulation of chronic inflammatory response
Neutrophil homeostasis
Negative regulation of t cell receptor signaling pathway
Negative regulation of antigen receptor mediated signaling pathway
Natural killer cell cytokine production
Angiogenesis involved in wound healing
Regulation of synaptic vesicle recycling
Cellular hyperosmotic response
Negative regulation of macrophage differentiation
P0
P14
M3
M12
M24
F
Lamina propria
Timepoint
Non canonical wnt signaling pathway
Notch signaling pathway
Positive regulation of canonical wnt signaling pathway
Positive regulation of non canonical wnt signaling pathway
Positive regulation of wnt signaling pathway
Regulation of canonical wnt signaling pathway
Regulation of notch signaling pathway
Response to bmp
Negative regulation of bmp signaling pathway
Regulation of bmp signaling pathway
Epithelial mesenchymal cell signaling
Mesenchymal epithelial cell signaling
Cell cycle dna replication
Positive regulation of stem cell proliferation
Cell cell signaling by wnt
Cell fate specification
Neutrophil activation involved in immune response
Macrophage activation
Macrophage migration
Macrophage proliferation
Stem cell proliferation
Mechanosensory behavior
Chronic inflammatory response
Activation of nf kappab inducing kinase activity
Inflammatory response to antigenic stimulus
Neuroinflammatory response
Positive regulation of inflammatory response
Macrophage inflammatory protein 1 alpha production
Inflammatory cell apoptotic process
Negative regulation of keratinocyte differentiation
Keratinocyte differentiation
Innate immune response in mucosa
Regulation of immune effector process
P0
P14
M3
M12
M24
Expression
−2
−1
0
1
2
