# Supplementary material for: Molecular Mechanisms of Age‐Related Taste Dysfunction Deciphered by Spatiotemporal Transcriptomics
Source: Aging Cell. 2026 Aug 20;25(9):e70674. doi: 10.1111/acel.70674 (PMC13494364; doi:10.1111/acel.70674)
Supplement: Supplementary file 3 [file ACEL-25-e70674-s003.pdf]

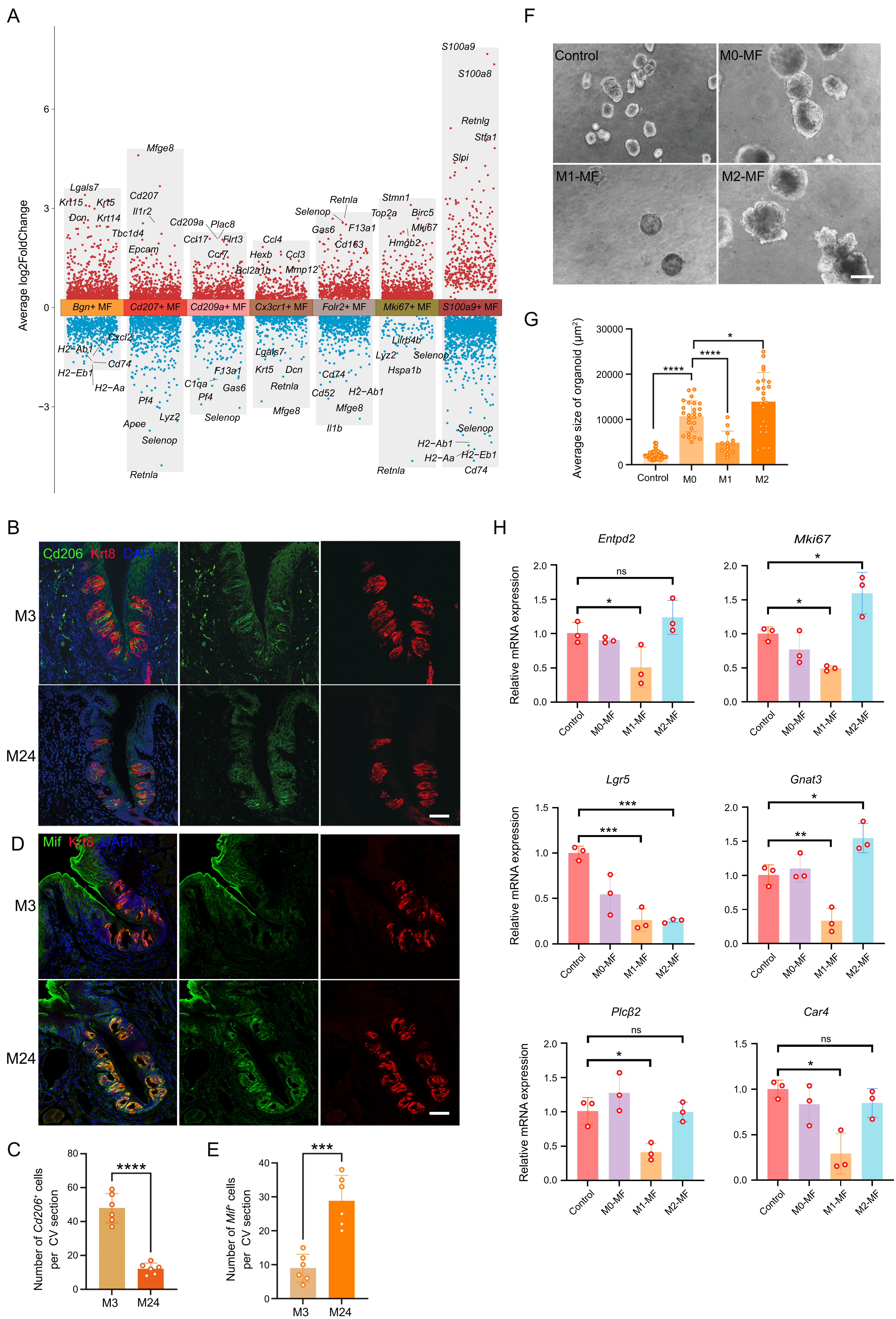

A
Average log2FoldChange
Bgn+ MF
Cd207+ MF
Cd209a+ MF
Cx3cr1+ MF
Folr2+ MF
Mki67+ MF
S100a9+ MF
B
Cd206 Krt8 DAPI
M3
M24
C
Number of Cd206+ cells per CV section
D
Mif Krt8 DAPI
E
Number of Mif+ cells per CV section
F
Control
M0-MF
M1-MF
M2-MF
G
Average size of organoid (μm²)
Control
M0
M1
M2
H
Entpd2
Mki67
Lgr5
Gnat3
Plcβ2
Car4
Relative mRNA expression
