# Supplementary material for: Molecular Mechanisms of Age‐Related Taste Dysfunction Deciphered by Spatiotemporal Transcriptomics
Source: Aging Cell. 2026 Aug 20;25(9):e70674. doi: 10.1111/acel.70674 (PMC13494364; doi:10.1111/acel.70674)
Supplement: Supplementary file 4 [file ACEL-25-e70674-s008.pdf]

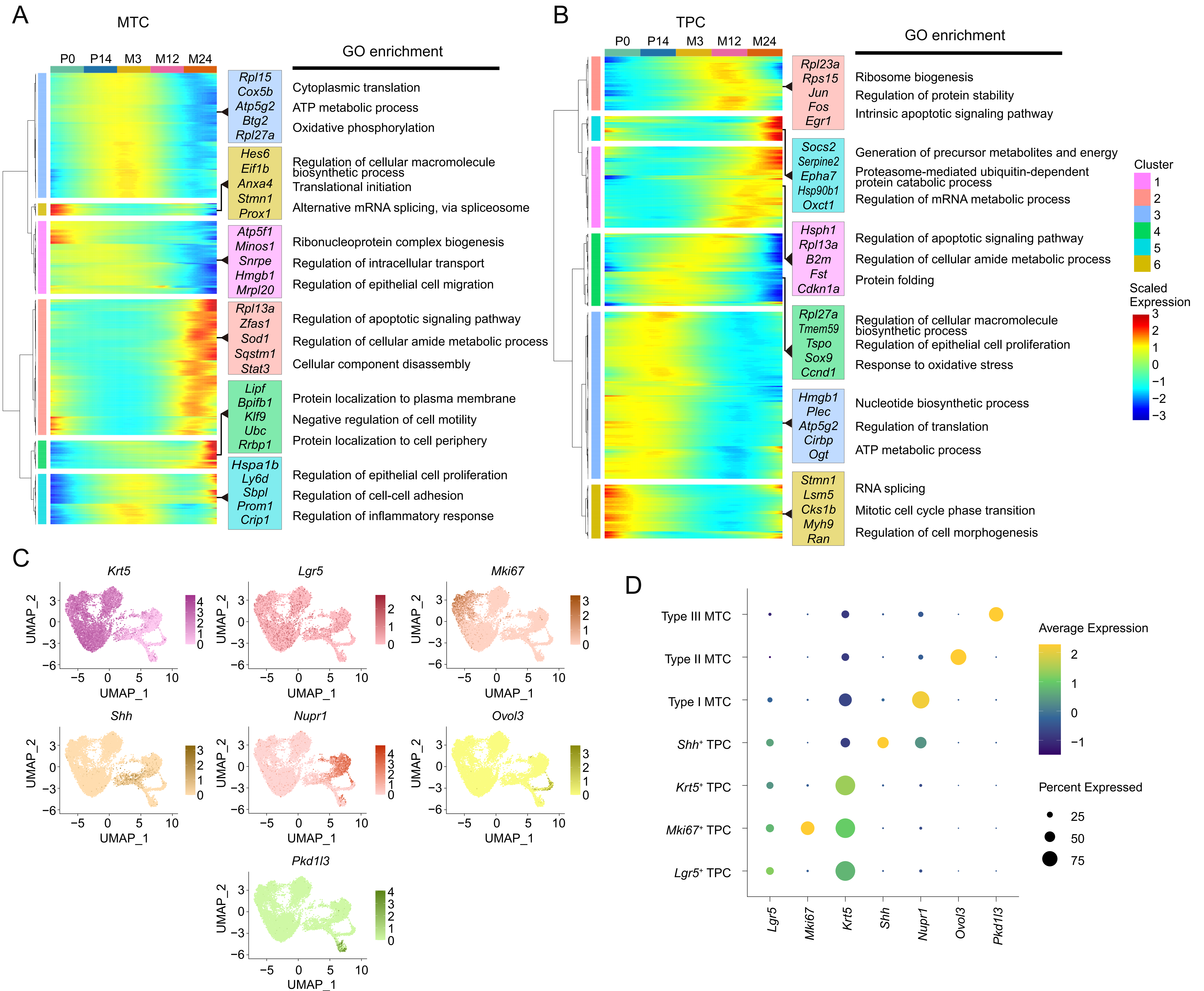
A
MTC
P0 P14 M3 M12 M24
GO enrichment
Rpl15 Cox5b Atp5g2 Btg2 Rpl27a
Cytoplasmic translation
ATP metabolic process
Oxidative phosphorylation
Hes6 Eif1b Anxa4 Stmn1 Prox1
Regulation of cellular macromolecule biosynthetic process
Translational initiation
Alternative mRNA splicing, via spliceosome
Atp5f1 Minos1 Snrpe Hmgb1 Mrpl20
Ribonucleoprotein complex biogenesis
Regulation of intracellular transport
Regulation of epithelial cell migration
Rpl13a Zfas1 Sod1 Sqstm1 Stat3
Regulation of apoptotic signaling pathway
Regulation of cellular amide metabolic process
Cellular component disassembly
Lipf Bpifb1 Klf9 Ubc Rrbp1
Protein localization to plasma membrane
Negative regulation of cell motility
Protein localization to cell periphery
Hspa1b Ly6d Sbpl Prom1 Crip1
Regulation of epithelial cell proliferation
Regulation of cell-cell adhesion
Regulation of inflammatory response
B
TPC
P0 P14 M3 M12 M24
GO enrichment
Rpl23a Rps15 Jun Fos Egr1
Ribosome biogenesis
Regulation of protein stability
Intrinsic apoptotic signaling pathway
Socs2 Serpine2 Epha7 Hsp90b1 Oxct1
Generation of precursor metabolites and energy
Proteasome-mediated ubiquitin-dependent protein catabolic process
Regulation of mRNA metabolic process
Hsph1 Rpl13a B2m Fst Cdkn1a
Regulation of apoptotic signaling pathway
Regulation of cellular amide metabolic process
Protein folding
Rpl27a Tmem59 Tspo Sox9 Ccnd1
Regulation of cellular macromolecule biosynthetic process
Regulation of epithelial cell proliferation
Response to oxidative stress
Hmgb1 Plec Atp5g2 Cirbp Ogt
Nucleotide biosynthetic process
Regulation of translation
ATP metabolic process
Stmn1 Lsm5 Cks1b Myh9 Ran
RNA splicing
Mitotic cell cycle phase transition
Regulation of cell morphogenesis
Cluster
1 2 3 4 5 6
Scaled Expression
3 2 1 0 −1 −2 −3
C
Krt5
Lgr5
Mki67
Shh
Nupr1
Ovol3
Pkd1l3
UMAP_1
UMAP_2
D
Type III MTC
Type II MTC
Type I MTC
Shh⁺ TPC
Krt5⁺ TPC
Mki67⁺ TPC
Lgr5⁺ TPC
Lgr5 Mki67 Krt5 Shh Nupr1 Ovol3 Pkd1l3
Average Expression
2 1 0 −1
Percent Expressed
25 50 75
