# Supplementary material for: Molecular Mechanisms of Age‐Related Taste Dysfunction Deciphered by Spatiotemporal Transcriptomics
Source: Aging Cell. 2026 Aug 20;25(9):e70674. doi: 10.1111/acel.70674 (PMC13494364; doi:10.1111/acel.70674)
Supplement: Supplementary file 5 [file ACEL-25-e70674-s010.pdf]

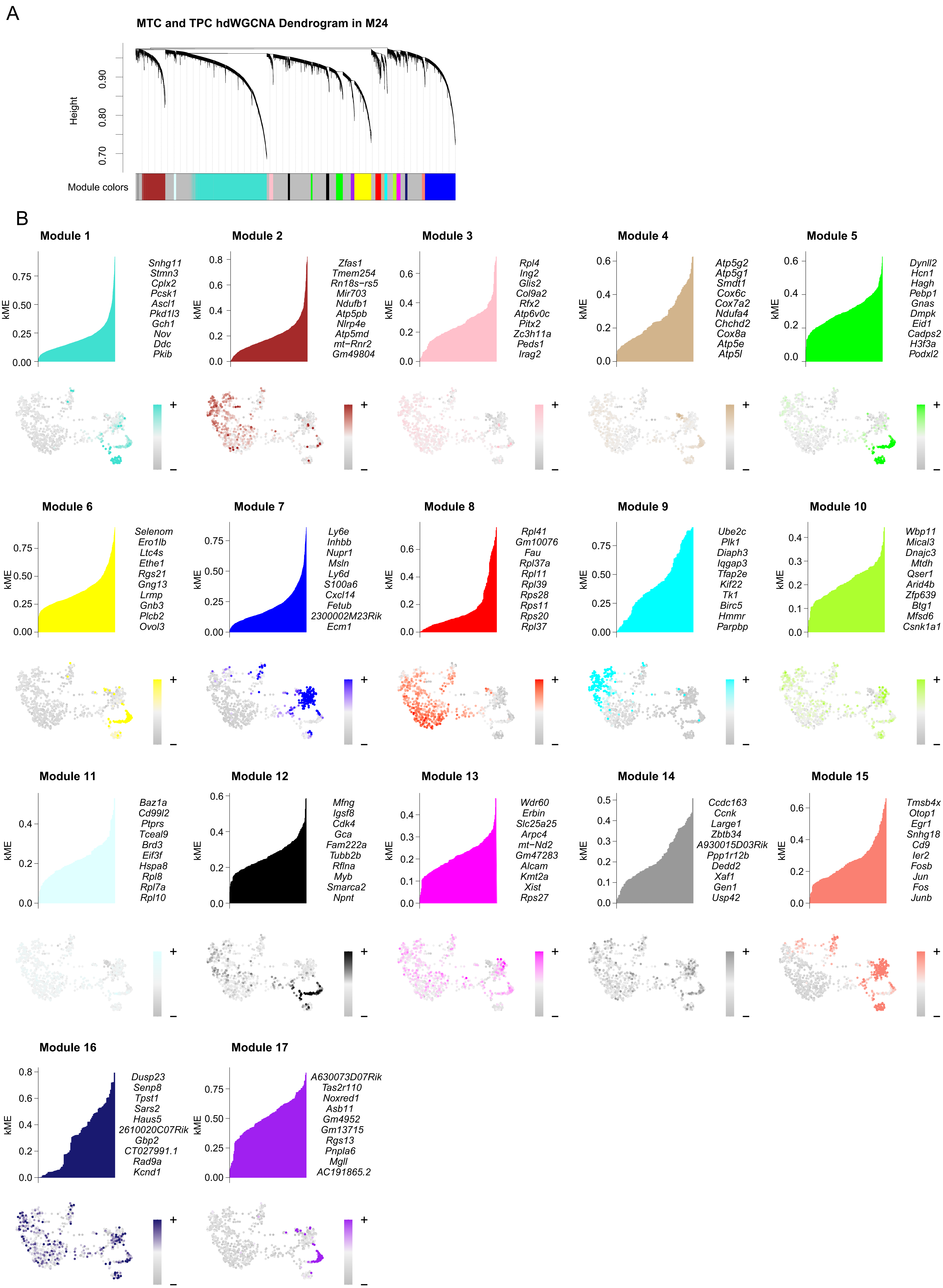
A
MTC and TPC hdWGCNA Dendrogram in M24
Height
0.70
0.80
0.90
Module colors
B
Module 1
kME
Snhg11
Stmn3
Cplx2
Pcsk1
Ascl1
Pkd1l3
Gch1
Nov
Ddc
Pkib
Module 2
Zfas1
Tmem254
Rn18s−rs5
Mir703
Ndufb1
Atp5pb
Nlrp4e
Atp5md
mt−Rnr2
Gm49804
Module 3
Rpl4
Ing2
Glis2
Col9a2
Rfx2
Atp6v0c
Pitx2
Zc3h11a
Peds1
Irag2
Module 4
Atp5g2
Atp5g1
Smdt1
Cox6c
Cox7a2
Ndufa4
Chchd2
Cox8a
Atp5e
Atp5l
Module 5
Dynll2
Hcn1
Hagh
Pebp1
Gnas
Dmpk
Eid1
Cadps2
H3f3a
Podxl2
Module 6
Selenom
Ero1lb
Ltc4s
Ethe1
Rgs21
Gng13
Lrmp
Gnb3
Plcb2
Ovol3
Module 7
Ly6e
Inhbb
Nupr1
Msln
Ly6d
S100a6
Cxcl14
Fetub
2300002M23Rik
Ecm1
Module 8
Rpl41
Gm10076
Fau
Rpl37a
Rpl11
Rpl39
Rps28
Rps11
Rps20
Rpl37
Module 9
Ube2c
Plk1
Diaph3
Iqgap3
Tfap2e
Kif22
Tk1
Birc5
Hmmr
Parpbp
Module 10
Wbp11
Mical3
Dnajc3
Mtdh
Qser1
Arid4b
Zfp639
Btg1
Mfsd6
Csnk1a1
Module 11
Baz1a
Cd99l2
Ptprs
Tceal9
Brd3
Eif3f
Hspa8
Rpl8
Rpl7a
Rpl10
Module 12
Mfng
Igsf8
Cdk4
Gca
Fam222a
Tubb2b
Rflna
Myb
Smarca2
Npnt
Module 13
Wdr60
Erbin
Slc25a25
Arpc4
mt−Nd2
Gm47283
Alcam
Kmt2a
Xist
Rps27
Module 14
Ccdc163
Ccnk
Large1
Zbtb34
A930015D03Rik
Ppp1r12b
Dedd2
Xaf1
Gen1
Usp42
Module 15
Tmsb4x
Otop1
Egr1
Snhg18
Cd9
Ier2
Fosb
Jun
Fos
Junb
Module 16
Dusp23
Senp8
Tpst1
Sars2
Haus5
2610020C07Rik
Gbp2
CT027991.1
Rad9a
Kcnd1
Module 17
A630073D07Rik
Tas2r110
Noxred1
Asb11
Gm4952
Gm13715
Rgs13
Pnpla6
Mgll
AC191865.2
+
−
