# Supplementary material for: Molecular Mechanisms of Age‐Related Taste Dysfunction Deciphered by Spatiotemporal Transcriptomics
Source: Aging Cell. 2026 Aug 20;25(9):e70674. doi: 10.1111/acel.70674 (PMC13494364; doi:10.1111/acel.70674)
Supplement: Supplementary file 6 [file ACEL-25-e70674-s011.pdf]

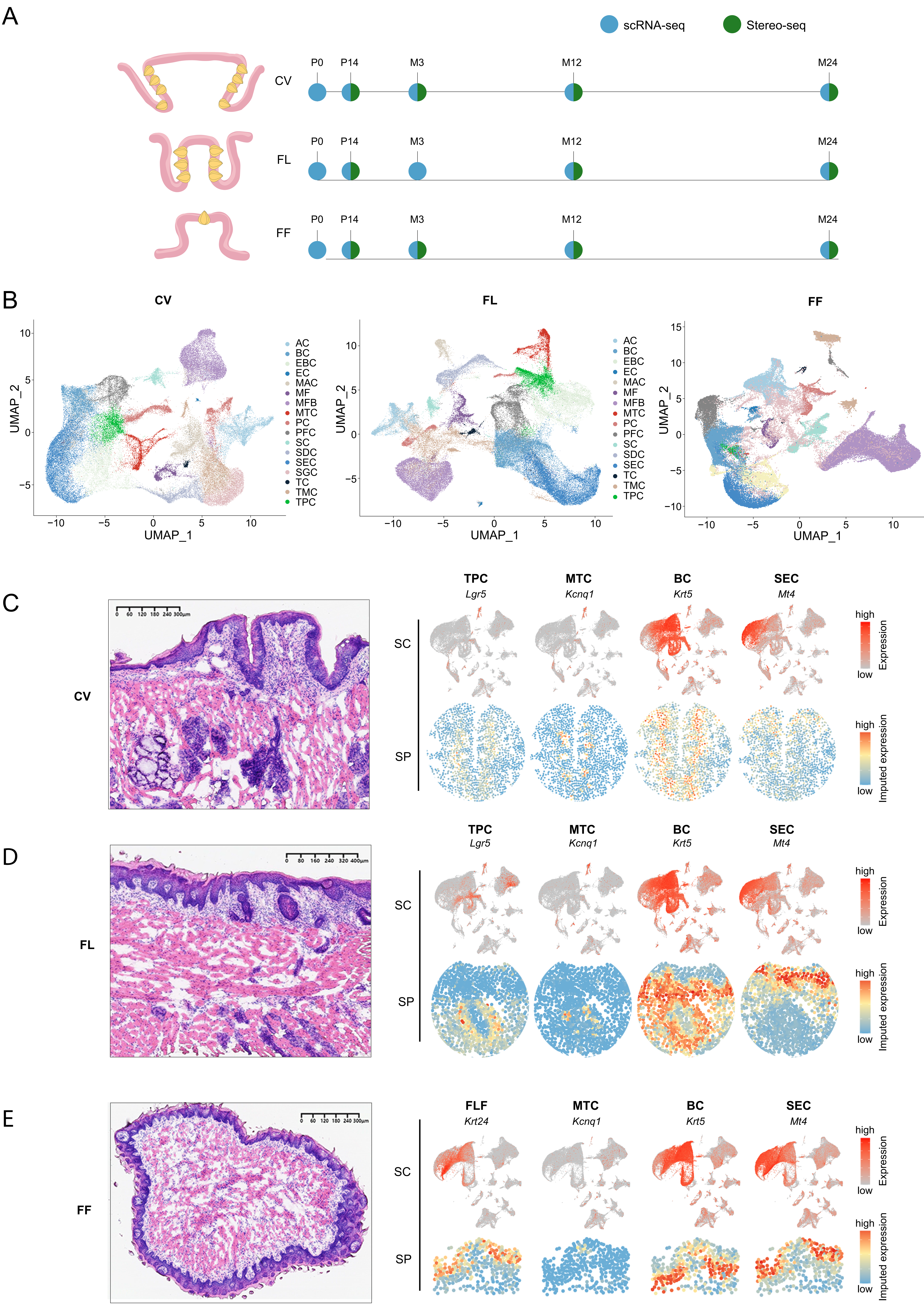
A
scRNA-seq
Stereo-seq
CV
FL
FF
P0
P14
M3
M12
M24
B
CV
FL
FF
UMAP_1
UMAP_2
AC
BC
EBC
EC
FLF
MAC
MF
MFB
MTC
PC
PFC
SC
SDC
SEC
SGC
TC
TMC
TPC
C
D
E
0 60 120 180 240 300μm
0 80 160 240 320 400μm
TPC
Lgr5
FLF
Krt24
MTC
Kcnq1
BC
Krt5
SEC
Mt4
SC
SP
high
low
Expression
Imputed expression
