# Supplementary material for: Molecular Mechanisms of Age‐Related Taste Dysfunction Deciphered by Spatiotemporal Transcriptomics
Source: Aging Cell. 2026 Aug 20;25(9):e70674. doi: 10.1111/acel.70674 (PMC13494364; doi:10.1111/acel.70674)
Supplement: Supplementary file 7 [file ACEL-25-e70674-s015.docx]

**Supplementary Figures for**

**Molecular Mechanisms of Age-Related Taste Dysfunction Deciphered by Spatiotemporal Transcriptomics**

Wandong Zhao^1,5,8,#^, Xudong Cha^2,#^, Yingqi Xie^2,#^, Enhao Wang^2,#^, Wenlu Xing^5,8^, Zengyi Xu^2^, Langchao Liang^7,9^, Changhai Xiong^2^, Morong Zhou^1,5^, Penghui Fu^2^, Zhe Wang^2^, Xin Liu^8^, Yiqun Yu^3,4,*^, Huanhai Liu^2,*^, Hanbo Li^5,6,8,*^, Wenwen Ren^2,*,†^

^1^ College of Life Sciences, University of Chinese Academy of Sciences, Beijing 10049, China.

^2^ Department of Otolaryngology, the Second Affiliated Hospital of the Naval Military University (Shanghai Changzheng Hospital), Shanghai, People's Republic of China.

^3^ Ear, Nose & Throat Institute, Department of Otolaryngology, Eye, Ear, Nose & Throat Hospital, Fudan University, Shanghai 200031, People's Republic of China.

^4^ Olfactory Disorder Diagnosis and Treatment Center, Eye, Ear, Nose & Throat Hospital, Fudan University, Shanghai 200031, People's Republic of China.

^5^ BGI Research, Qingdao 266555, China.

^6^ State Key Laboratory of Genome and Multi-omics Technologies, BGI Research, Shenzhen 518083, China.

^7^ BGI Research, Hangzhou 310030, China.

^8^ BGI Research, Shenzhen 518083, China.

^9^ School of Biology and Biological Engineering, South China University of Technology, Guangzhou, 510006, China.

**^†^ Lead contact:**

Wenwen Ren: [wenwenren@smmu.edu.cn](mailto:wenwenren@smmu.edu.cn)

**^*^ Corresponding author:**

Yiqun Yu: [yu_yiqun@fudan.edu.cn](mailto:yu_yiqun@fudan.edu.cn)

Huanhai Liu: [liuhuanhaiok@smmu.edu.cn](mailto:liuhuanhaiok@smmu.edu.cn)

Hanbo Li: lihanbo@genomics.cn

Wenwen Ren: [wenwenren@smmu.edu.cn](mailto:wenwenren@smmu.edu.cn)

**^#^ These authors contributed equally to this work.**

**Data and Code availability**

All data needed to evaluate the conclusions in the paper are present in the paper and/or the Supplementary Materials. Raw sequencing data generated in this study were deposited at CNGB Nucleotide Sequence Archive (accession code: CNP0007410). All original codes supporting this study were hosted on GitHub: <https://github.com/Zhaowado/Inflammatory-aging-taste>. Requests for the data should be submitted to the lead contact W. Ren..

**Funding information**

This work was supported by National Natural Science Foundation of China Grants (82271136 to W.R., U23A20439 to H.L., 82571283 and 32271044 to Y.Y.); Science and Technology Commission of Shanghai Municipality (23ZR1409600 to Y.Y.); Shanghai Municipal Education Commission, the Shanghai Eastern Scholar Program (GZ2022006 to Y.Y.); Shenzhen Science and Technology Program (RCJC20221008092804002 to H.L.).

**Contributions**

W.R., H.L., H.L., and Y.Y., designed the research; W.Z., Y.Y., W.X., Z.X., L.L., and M.Z. analyzed the data; X.C., Y.X., and E.W. performed the research; C.X., P.F., and Z.W. performed the statistical analyses; X.L. contributed to the interpretation of macrophage-related experimental results; W.R., W.Z., Y.Y., and H.L., wrote the paper.

**Acknowledgements**

We thank the staff members of the Integrated Laser Microscopy System at the National Facility for Protein Science in Shanghai (NFPS), Zhangjiang Lab, China for providing technical support and assistance in data collection and analysis.

**Conflict of interest disclosure**

The authors declare that they have no competing interests.

**Abstract**

**Age-related taste dysfunction impairs nutrition and quality of life, contributing to metabolic disorders and frailty in the elderly, yet its cellular and molecular basis remains poorly** **understood. By integrating single-cell RNA and spatial transcriptomics sequencing, we systematically mapped murine taste bud aging across five developmental stages from neonatal to aged. Our findings reveal that age-associated taste impairment is driven by the progressive depletion of stem/progenitor cell differentiation capacity, predominantly characterized in the posterior tongue, and mediated by inflamm-aging responses and micro-environmental alterations. Specifically, we identified macrophage infiltration and polarization shifts in aged circumvallate and foliate papillae, underscoring the crucial role of macrophages in age-related taste decline.** **A co-culture model using taste organoids with conditioned media from polarized macrophage revealed that M1-polarized cells suppress taste organoid proliferation and differentiation. Mechanistically, this inhibition may be mediated by macrophage-derived Thbs1, as recombinant Thbs1 treatment directly suppressed taste progenitor cell expansion in vitro. Together, this study provides the first comprehensive spatiotemporal atlas of taste bud aging and identifies novel therapeutic targets for the treatment of age-related taste disorders.**

**Key words: taste, aging, taste progenitor cell, macrophage, organoids**

Figure S1. Spatially resolved single-cell transcriptomic atlas of mouse taste papillae.

(A) Experimental timeline and multi-omics strategy for profiling three types of taste papillae. (B) UMAP visualization of integrated scRNA-seq (SC) and Stereo-seq (SP) datasets per tissues, with cell identity labels transferred from scRNA-seq annotations. (C-E) Left: HE staining of full sagittal sections of CV, FL and FF papillae. Right: Spatial expression patterns of molecular markers; the top subpanels display the SC signals of selected markers in each tissue, while the bottom subpanels present the SP expression patterns of the same gene set.

­

Figure S2. Cell identify marker expression and age associated pathway remodeling across three tissues.

(A-C) Violin plots showing the expression of representative marker genes across five stages in three major taste papilla compartments: non-keratinized epithelium, including TPC, MTC, MAC, and SDC; keratinized epithelium, including BC, EBC, FLF, PFC, SEC, and SGC; and lamina propria, including AC, EC, MF, MFB, PC, SC, and TC. (D-F) Heatmaps showing GSVA analysis scores for selected GO terms across five stages in non-keratinized epithelium, keratinized epithelium, and lamina propria.

Figure S3. Gene expression patterns in taste cell subtypes.

(A, B) Heatmaps of pseudotime-dependents DEGs in MTC and TPC, grouped into six expression patterns. Corresponding representative genes and enriched GO terms are displayed in middle and right panels, respectively. (C) Gene expression plot displaying the molecular markers for specific taste cell subtypes; colors intensity indicates expression level. (D) Dot plot showing average expression of cell-type-specific genes across MTC and TPC subtypes. Dot color represents expression level, and dot size reflects percentage of cells expressing the gene within each cluster.

Figure S4. Comparative analysis of MF polarization and functional effects between young and aged taste papillae (related to Figure 5).

(A) DEGs across MF subtypes, with red marking upregulation and blue indicating downregulation. (B, C) Immunofluorescence staining (Cd206, Krt8) and quantification of Cd206^+^ cells in CV from M3 and M24 mice. (D, E) Immunofluorescence staining (Mif, Krt8) and quantification of Mif^+^ cells in CV from M3 and M24 mice. (F) Bright-field images of taste organoids co-cultured with conditioned media from polarized MF. (G) Quantification of average size of taste organoids under differentiate conditions. (H) RT-qPCR analysis of mRNA expression levels of taste cell lineage, stemness, and proliferation markers in CV-derived taste organoids treated with control medium or conditioned medium from M0-, M1-, and M2- MF. *p<0.05, ***p < 0.001, ****p < 0.0001 (unpaired t-test). Scale bars: 50 μm in (B), 100 μm in (F).

Figure S5. Cell-cell communication analysis between MF and taste cell subtypes at M24.

(A) Dot plot displaying all ligand-receptor pairs involved in MF-taste cell subtypes interactions, as inferred by CellChat. (B) Circle plot showing outgoing interaction strength from MF subtypes. Edge width represents the communication weight, and circle size reflects the number of cells per group.

Figure S6. hdWGCNA analysis showing the gene co-expression network between MTC and TPC in M24.

(A) Dendrogram of 17 co-expression modules identified in taste cells. Nodes represent genes clustered by expression similarity (branch height indicates co-expression relatedness). Colored bars denote distinct gene modules. (B) Density curve plot (Upper) showing the module membership ranked by kME. The top 10 genes with highest kME values per module are listed on the right. Bottom: UMAP plot mapping the gene expression patterns in each module, with gray indicating low expression and color intensity reflecting expression strength.

Table S1. (separate file)

Cell number summary across taste papillae, time points, and cell types.

Table S1A. The total number of cells per time point per taste papillae.

Table S1B. The total number of taste cells per time point per taste papillae.

Table S1C. The total number of cell types per time point per taste papillae.

Table S2. (separate file)

GO term of taste sense.

Table S3. (separate file)

Gene sets for TPC scoring.

Table S4. (separate file)

DEGs and GO enrichment between M24 and other timepoints across MTC and TPC.

Table S4A. DEGs between M24 and other time points across MTC and TPC.

Table S4B. GO enrichment of corresponding DEGs in MTC vs TPC at M24 and other time points.

Table S5. (separate file)

Gene list for scoring analysis.

Table S5A. Gene list for inflammatory response scoring analysis.

Table S5B. Gene list for SASP scoring analysis.

Table S6. (separate file)

The target genes predicted by Nichenet.

Table S7. (separate file)

DEGs of each taste subtype between M24 and other time points

Table S8. (separate file)

Antibodies used for immunofluorescence.

Table S8A. Primary antibodies for immunofluorescence.

Table S8B. Secondary antibodies for immunofluorescence.
